# The Morelloid clade of *Solanum* L. (Solanaceae) in Argentina: nomenclatural changes, three new species and an updated key to all taxa

**DOI:** 10.3897/phytokeys.164.54504

**Published:** 2020-10-21

**Authors:** Sandra Knapp, Franco Chiarini, Juan J. Cantero, Gloria E. Barboza

**Affiliations:** 1 Department of Life Sciences, Natural History Museum, Cromwell Road, London SW7 5BD, UK Natural History Museum London United Kingdom; 2 Museo Botánico, IMBIV (Instituto Multidisciplinario de Biología Vegetal), Universidad Nacional de Córdoba, Casilla de Correo 495, 5000, Córdoba, Argentina Universidad Nacional de Córdoba Córdoba Argentina; 3 Departamento de Biología Agrícola, Facultad de Agronomía y Veterinaria, Universidad Nacional de Rio Cuarto, Ruta Nac. 36, km 601, 5804, Río Cuarto, Córdoba, Argentina Universidad Nacional de Rio Cuarto Río Cuarto Argentina

**Keywords:** Andes, black nightshades, dry forests, endemic, glandular pubescence, identification, new species, rarity

## Abstract

Since the publication of the Solanaceae treatment in “Flora Argentina” in 2013 exploration in the country and resolution of outstanding nomenclatural and circumscription issues has resulted in a number of changes to the species of the Morelloid clade of *Solanum* L. (Solanaceae) for Argentina. Here we describe three new species: *Solanum hunzikeri* Chiarini & Cantero, **sp. nov.**, from wet high elevation areas in Argentina (Catamarca, Salta and Tucumán) and Bolivia (Chuquisaca and Tarija), *S. marmoratum* Barboza & S. Knapp, **sp. nov.**, from central Argentina in Catamarca, La Pampa, La Rioja, San Juan and San Luis, and *S. tiinae* Barboza & S. Knapp, **sp. nov.**, from the mountains of Jujuy, La Rioja, Salta and Tucumán. We provide descriptions, illustrations and distribution maps for all new taxa. A table of nomenclatural changes and additional taxa now known to occur in Argentina summarizes additions and changes since the “Flora Argentina”. We also provide an updated key, including all new taxa for the country, to facilitate identification and further exploration.

## Introduction

*Solanum* L., with 1,400 species, is one of the largest genera of flowering plants (Frodin 2004). Its species occur worldwide, with highest diversity in South America, and in a wide variety of habitats, from deserts to tropical rainforests to high elevation grasslands. The genus comprises 13 major clades, one of which, the Leptostemonum clade or spiny solanums, contains approximately half the species. The non-spiny solanums are a paraphyletic grade (Särkinen et al. 2013) within which several monophyletic groups are resolved (Särkinen et al. 2013); one of these is the Morelloid clade (see Särkinen et al. 2015). Members of the clade are usually herbs or small short-lived subshrubs and the group is sister to the Dulcamaroid clade, a group of woody vines (See Knapp 2013). Species of the Morelloid clade are found worldwide and are being treated in a series of monographs (e.g., Old World taxa in Särkinen et al. 2018; Caribbean, North and Central American taxa in Knapp et al. 2019; South American taxa in G.E. Barboza et al., in prep); by far the highest diversity occurs in western South America (Särkinen et al. 2015). Of the 62 species of South American morelloids, 38 species are found in Argentina with 8 species occurring as country endemics (Table 1; 37 with 7 endemics excluding *S. concarense* Hunz., see below), making the country a hotspot for morelloid diversity. It is equalled only by Bolivia also with 38 species, 21 of which are in common with Argentina.

**Table 1.** Members of the Morelloid clade occurring in Argentina with their treatment in *Flora Argentina* (Barboza et al. 2013) and the reasons for changes here. Circumscription changes and nomenclatural details will be treated in full in the upcoming monograph (G.E. Barboza et al. in prep.). Country endemics are in **bold face** type. NB: *Solanum concarense* (*) is included for consistency with *Flora Argentina* (Barboza et al. 2013), although it has been shown to belong to the Dulcamaroid (see text) rather than the Morelloid clade.

Species recognised here	Treatment in Flora Argentina (Barboza et al. 2013)	Reason for change
*Solanum aloysiifolium* Dunal	same	
*Solanum americanum* Mill.	same	
***Solanum annuum* C.V.Morton**	same	
*Solanum caesium* Griseb.	same	
*Solanum chenopodioides* Lam.	same	
*Solanum cochabambense* Bitter	as synonym of *S. aloysiifolium* Dunal	new circumscription based on examination of material from northern South America
***Solanum concarense* Hunz.** *	same (included here for continuity)	now placed as a member of the Dulcamaroid clade (see text)
*Solanum echegarayi* Hieron.	same	new circumscription, now includes *S. hastatilobum* Bitter
*Solanum fiebrigii* Bitter	same	
*Solanum furcatum* Dunal	same	
*Solanum gilioides* Rusby	same	
***Solanum glandulosipilosum* Bitter**	same	
*Solanum grandidentatum* Phil.	as *S. excisirhombeum* Bitter	Older name (nomenclatural change)
*Solanum huayavillense* Del Vitto & Peten.	same	
*Solanum hunzikeri* Chiarini & Cantero	not included	described here
***Solanum marmoratum* Barboza & S. Knapp**	not included	described here
*Solanum michaelis* Särkinen & S. Knapp	not included	new distribution record based on *Kiesling 8354* (CORD, SI)
*Solanum nitidibaccatum* Bitter	same	
*Solanum palitans* C.V.Morton	same	
*Solanum paucidens* Bitter	not included	new distribution record based on *Johnson 843* (CORD)
*Solanum physalidicalyx* Bitter	as *S. tweedianum* Hook.	see text
*Solanum physalifolium* Rusby	same	
*Solanum pilcomayense* Morong	same	
***Solanum profusum* C.V.Morton**	same	
*Solanum pygmaeum* Cav.	same	
***Solanum riojense* Bitter**	as synonym of *S. echegarayi* Hieron.	new circumscription based on examination of more material
***Solanum salamancae* Hunz. & Barboza**	same	
*Solanum salicifolium* Phil.	treated as member of the Dulcamaroid clade	new phylogenetic position as member of the Morelloid clade clarified (Särkinen et al. 2015)
*Solanum sarrachoides* Sendtn.	same	
*Solanum sinuatiexcisum Bitter*	same	
*Solanum sinuatirecurvum* Bitter	same	
***Solanum tiinae* Barboza & S. Knapp**	not included	described here
*Solanum triflorum Nutt.*	same	
*Solanum tripartitum* Dunal	same	
*Solanum tweedieanum* Hook.	as *S. atriplicifolium* Gillies ex Nees	see text
*Solanum weddellii* Phil.	as *S. chamaesarachidium* Bitter	older name (nomenclatural change)
*Solanum woodii* Särkinen & S. Knapp	not included	new distribution record based on *Nee & Bohs 50823* (NY)
*Solanum zuloagae* Cabrera	same	

Solanaceae were treated in the multi-volume “Flora Argentina” in 2013 (Anton and Zuloaga 2013), and the treatment of the Morelloid clade recognised 30 species (Grupo VII. Moreloide; Barboza et al. 2013). Further exploration of the country and herbaria, coupled with taxonomic and nomenclatural work as part of the monograph, has resulted in an additional seven species for the country, and name changes for three species treated in the *Flora* (see Table 1). Here we document changes, describe new taxa and provide a revised key and provincial distribution (Table 2) for all morelloid species in the county.

**Table 2.** Morelloid species occurring in each province of Argentina (specimens seen by the authors, see Suppl. materials 1, 2 and NHM Data Portal, https://doi.org/10.5519/0062836). No morelloid species have been collected from Tierra del Fuego or Antarctica. *Solanum concarense* is included for consistency with *Flora Argentina* (Barboza et al. 2013), although it has been shown to belong to another clade (see text). NB: *Solanum concarense* (*) is included for consistency with *Flora Argentina* (Barboza et al. 2013), although it has been shown to belong to the Dulcamaroid (see text) rather than the Morelloid clade.

Province	*Solanum* species with records
Buenos Aires (incl. DF)	*S. americanum*, *S. chenopodioides*, *S. nitidibaccatum*, *S. palitans*, *S. pilcomayense*, *S. pygmaeum*, *S. sarrachoides*, *S. triflorum*, *S. tweedieanum*
Catamarca	*S. aloysiifolium*, *S. annuum*, *S. cochabambense*, *S. echegarayi*, *S. huayavillense*, *S. hunzikeri*, *S. marmoratum*, *S. nitidibaccatum*, *S. palitans*, *S. physalidicalyx*, *S. physalifolium*, *S. salamancae*, *S. salicifolium*, *S. sarrachoides*, *S. sinuatirecurvum*, *S. tweedieanum*, *S. weddellii*
Chaco	*S. aloysiifolium*, *S. americanum*, *S. caesium*, *S. chenopodioides*, *S. nitidibaccatum*, *S. pilcomayense*, *S. pygmaeum*, *S. sarrachoides*, *S. tweedieanum*
Chubut	*S. furcatum*, *S. nitidibaccatum*, *S. triflorum*
Córdoba	*S. aloysiifolium*, *S. americanum*, *S. chenopodioides*, *S. echegarayi*, *S. nitidibaccatum*, *S. palitans*, *S. physalidicalyx*, *S. pilcomayense*, *S. pygmaeum*, *S. salicifolium*, *S. triflorum*, *S. tweedieanum*
Corrientes	*S. americanum*, *S. chenopodioides*, *S. paucidens*, *S. pilcomayense*, *S. pygmaeum*
Entre Ríos	*S. americanum*, *S. chenopodioides*, *S. nitidibaccatum*, *S. pilcomayense*, *S. pygmaeum*, *S. salicifolium*, *S. sarrachoides*, *S. tweedieanum*
Formosa	*S. americanum*, *S. pilcomayense*, *S. tweedieanum*
Jujuy	*S. aloysiifolium*, *S. annuum*, *S. caesium*, *S. chenopodioides*, *S. cochabambense*, *S. fiebrigii*, *S. gilioides*, *S. glandulosipilosum*, *S. grandidentatum*, *S. huayavillense*, *S. michaelis*, *S. palitans*, *S. physalidicalyx*, *S. physalifolium*, *S. profusum*, *S. riojense*, *S. salicifolium*, *S. sinuatiexcisum*, *S. sinuatirecurvum*, *S. tiinae*, *S. tripartitum*, *S. tweedieanum*, *S. weddellii*, *S. woodii*
La Pampa	*S. chenopodioides*, *S. marmoratum*, *S. pygmaeum*, *S. salicifolium*, *S. triflorum*, *S. tweedieanum*
La Rioja	*S. aloysiifolium*, *S. chenopodioides*, *S. cochabambense*, *S. echegarayi*, *S. marmoratum*, *S. nitidibaccatum*, *S. physalidicalyx*, *S. riojense*, *S. salicifolium*, *S. tiinae*, *S. triflorum*, *S. tweedieanum*, *S. weddellii*
Mendoza	*S. americanum*, *S. chenopodioides*, *S. echegarayi*, *S. nitidibaccatum*, *S. salicifolium*, *S. sarrachoides*, *S. triflorum*, *S. tweedieanum*
Misiones	*S. americanum*, *S. paucidens*, *S. pilcomayense*
Neuquén	*S. furcatum*, *S. nitidibaccatum*, *S. pygmaeum*, *S. triflorum*
Río Negro	*S. chenopodioides*, *S. furcatum*, *S. nitidibaccatum*, *S. salicifolium*, *S. triflorum*, *S. tweedieanum*
Salta	*S. aloysiifolium*, *S. americanum*, *S. annuum*, *S. caesium*, *S. chenopodioides*, *S. cochabambense*, *S. echegarayi*, *S. fiebrigii*, *S. glandulosipilosum*, *S. huayavillense*, *S. hunzikeri*, *S. michaelis*, *S. nitidibaccatum*, *S. palitans*, *S. physalidicalyx*, *S. physalifolium*, *S. pilcomayense*, *S. profusum*, *S. riojense*, *S. salamancae*, *S. salicifolium*, *S. sarrachoides*, *S. sinuatiexcisum*, *S. sinuatirecurvum*, *S. tiinae*, *S. tripartitum*, *S. tweedieanum*, *S. weddellii*, *S. zuloagae*
San Juan	*S. echegarayi*, *S. marmoratum*, *S. nitidibaccatum*, *S. physalidicalyx*, *S. salicifolium*, *S. triflorum*, *S. tweedieanum*
San Luis	*S. aloysiifolium*, *S. chenopodioides*, *S. concarense* *, *S. echegarayi*, *S. marmoratum*, *S. nitidibaccatum*, *S. physalidicalyx*, *S. pygmaeum*, *S. salicifolium*, *S. sarrachoides*, *S. triflorum*, *S. tweedieanum*
Santa Cruz	*S. nitidibaccatum*, *S. triflorum*
Santa Fé	*S. americanum*, *S. chenopodioides*, *S. pilcomayense*, *S. pygmaeum*, *S. triflorum*
Santiago del Estero	*S. aloysiifolium*, *S. americanum*, *S. nitidibaccatum*, *S. physalidicalyx*, *S. pilcomayense*, *S. pygmaeum*, *S. sarrachoides*, *S. tweedieanum*
Tucumán	*S. aloysiifolium*, *S. americanum*, *S. annuum*, *S. chenopodioides*, *S. cochabambense*, *S. fiebrigii*, *S. gilioides*, *S. glandulosipilosum*, *S. huayavillense*, *S. hunzikeri*, *S. nitidibaccatum*, *S. palitans*, *S. physalidicalyx*, *S. pilcomayense*, *S. pygmaeum*, *S. riojense*, *S. salamancae*, *S. salicifolium*, *S. sinuatiexcisum*, *S. tiinae*, *S. triflorum*, *S. tweedieanum*, *S. weddellii*, *S. zuloagae*

## Materials and methods

Our species circumscriptions are based on revision of herbarium material accompanied by detailed examination of living plants in the field and, where possible, in cultivation at the Instituto Multidisciplinario de Biología Vegetal (IMBIV) in Córdoba, Argentina. We have also used published and unpublished results from molecular phylogenetic study of the entire Morelloid clade (Särkinen et al. 2015; R. Hilgenhof, pers. comm.) to include or exclude taxa from the group. Descriptions for the new species are based on specimens from 14 herbaria (acronyms follow Index Herbariorum, http://sweetgum.nybg.org/science/ih/): BAA, BAB, BM, BR, CORD, CTES, E, G, K, LIL, MO, SI, US, W. Many more herbaria have been consulted during the course of monographic work on the Morelloid clade; these will be listed in full in the upcoming monograph, and details of all specimens seen to date from Argentina can be found in the Suppl. materials 1, 2: (SM 1, all morelloid species; SM 2, the three new species described here) and on the NHM Data Portal (https://doi.org/10.5519/0062836).

Measurements were made from dried herbarium material supplemented by measurements and observations from living and cultivated material. Colours (e.g., corollas, fruits, etc.) are described from living material or from herbarium label data. Specimens with latitude and longitude data on the labels were mapped directly. Some species had few or no georeferenced collections; in these cases we retrospectively georeferenced the collections using available locality data. Maps were constructed with the points in the centres of degree squares in a 1° square grid. Conservation threat status was assessed following the IUCN Red List Categories and Criteria (IUCN 2019) using the GIS-based method (Bachman et al. 2011) as implemented in the online assessment tools in GeoCat (http://geocat.kew.org). The Extent of Occurrence (EOO) measures the range of the species, and the Area of Occupancy (AOO) represents the number of occupied points within that range based on the default grid size of 2 km^2^. We have given more weight to the EOO in the threat assessments for relatively widespread species; AOO is very sensitive to georeferencing bias and collecting effort.

## Taxonomic treatment

### Name changes for Morelloid species in Argentina

Changes for inclusion and nomenclature for morelloids since the publication of “Flora Argentina” (Barboza et al. 2013) are summarised in Table 1, but some comment is necessary here. The widespread and highly variable species *S. salicifolium* Phil. was treated as a member of the Dulcamaroid clade by Knapp (2013) and in the *Flora*, but further work with DNA sequence data places *S. salicifolium* nested amongst the “Black nightshade” group of Särkinen et al. (2015). Further analyses have supported this position (E. Gagnon et al., pers. comm.). *Solanum salicifolium* is included here in the key for clarity, even though it was included in the Dulcamaroid clade earlier (Knapp 2013).

*Solanum concarense* was included as a member of the clade in Barboza et al. (2013), but subsequent phylogenetic analysis (R. Hilgenhof, pers. comm.) has revealed that it instead is nested within the Dulcamaroid clade. We include it here in the key for clarity, but it will not be treated as a member of the group in the upcoming monograph and a full species description is available on Solanaceae Source (http://solanaceaesource.org).

Several names have changed due to the clarification and subsequent resurrection (Särkinen et al. 2015) of older names coined by R.A. Philippi for species from high elevation areas of Chile adjacent to Argentina (Philippi 1891); *S. weddellii* Phil. is the older name for what was previously recognised (Barboza 2004) as *S. chamaesarachidium* Bitter and *S. grandidentatum* Phil. for the taxon previously recognised (Edmonds 1972) as *S. excisirhombeum* Bitter. These names have been in use since Särkinen et al. (2015). *Solanum cochabambense* Bitter was treated as a synonym of *S. aloysiifolium* Dunal in Barboza et al. (2013), but subsequent study through the entire range of *S. cochabambense* (north to Peru) has shown the two taxa to be distinct; we therefore recognise them as separate here.

### Re-evaluation of synonymy in two common glandular-pubescent taxa

**Figure 1.**
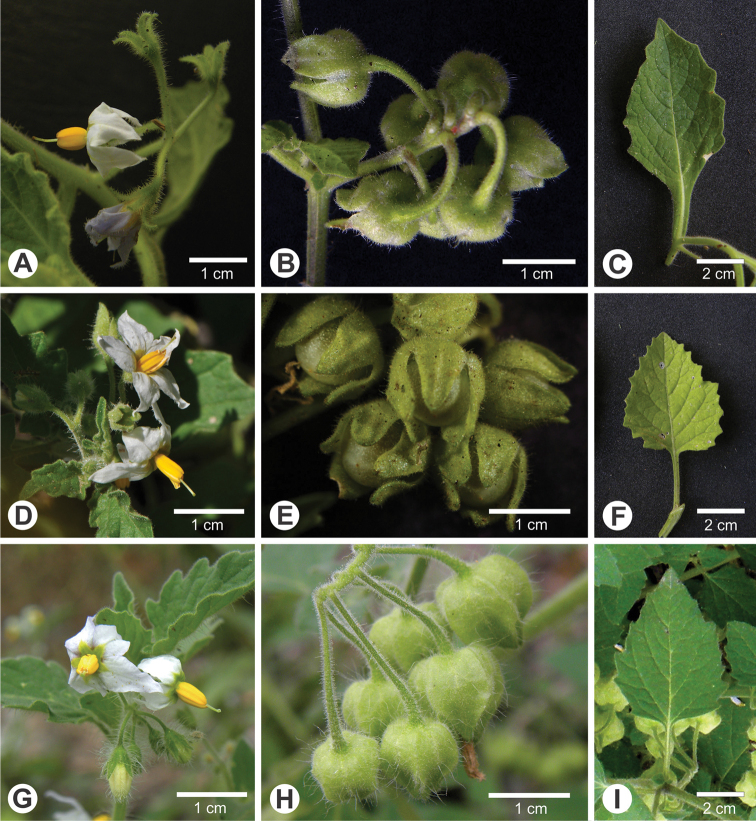
*Solanum hunzikeri* Chiarini & Cantero (**A–C**) compared to *S. tweedieanum* Hook. (**D–F**) and *S. physalidicalyx* Bitter (**G–I**) **A** habit (*Barboza et al. 4763*) **B** calyx morphology of developing fruits (*Barboza et al. 4763*) **C** leaf from mature stem (*Barboza et al. 4763*) **D** habit (*Barboza et al. 3496*) **E** calyx morphology of developing fruits (*Barboza et al. 4798*) **F** leaf from mature stem (*Barboza et al. 4798*) **G** habit (*Barboza et al. 3983*) **H** calyx morphology of developing fruits (*Barboza et al. 3983*) **I** leaf from mature stem (*Barboza et al. 3983*). Photos **A–C, E, F** by M. Gritti, **D** by S. Knapp, **G–I** by G.E. Barboza.

Re-evaluation of taxon circumscription and types for the upcoming monograph has revealed that two names for species with glandular trichomes and accrescent calyces were previously incorrectly applied in “Flora Argentina” and elsewhere (Barboza et al. 2013; Särkinen and Knapp 2016). In Barboza et al. (2013), two taxa were recognised, *S.* “*tweedianum*” Hook. (a mis-spelling of *S. tweedieanum*, see below) and *S. atriplicifolium* Gillies ex Nees, both of which are glandular-pubescent with ovate, shallowly toothed leaves. *Solanum physalidicalyx* Bitter, the name recognised here for a distinct species with highly inflated calyces, was erroneously put into synonymy with *S. tweedieanum*; the type of *S. tweedieanum* does not match these specimens but is a better match for plants called *S. atriplicifolium* in 2013. The type of *S. tweedieanum* comes from a plant cultivated at Kew that was collected in flower only; it lacks the diagnostic calyx characters (see Fig. 1 and the key presented here) that enable easy identification in this group, but anther length can also be used to distinguish those plants not in fruit. Plants with inflated calyces have shorter anthers than do those with calyces that are merely accrescent and tightly investing the berry; the types of both *S. tweedieanum* and *S. atriplicifolium* have longer (to 6 mm) anthers and belong to the same species, for which the oldest name is *S. tweedieanum*. We present here a revised synonymy for the two species of glandular-pubescent morelloids with anthers more than 3 mm long that occur in Argentina to correct the error in “Flora Argentina” (Barboza et al. 2013).

#### Solanum physalidicalyx

Taxon classificationPlantaeSolanalesSolanaceae

Bittter, Repert. Spec. Nov. Regni Veg. 11: 212. 1912

5C355DF2-DB58-5359-89D0-DD017F2E982F

Fig. 1G–I

Solanum physalidicalyx Bitter var. integrascens Bitter, Repert. Spec. Nov. Regni Veg. 11: 213. 1912. Type. Argentina. Salta: [Dtto. La Caldera], Pasaje del Río Juramento, *P.G. Lorentz & G. Hieronymus [no number cited*] (no explicit type material located; likely homotypic with species).
Solanum physalidicalyx Bitter var. plurilobulatum Bitter, Repert. Spec. Nov. Regni Veg. 11: 213. 1912. Type. Argentina. Salta: [Dtto. La Caldera], Pasaje del Río Juramento, *P.G. Lorentz & G. Hieronymus [no number cited*] (no explicit type material located; likely homotypic with species).

##### Type.

Argentina. Salta: [Dtto. La Caldera], Pasaje del Río Juramento, Feb 1873, *P.G. Lorentz & G. Hieronymus 364* (holotype: B [destroyed]; lectotype, designated by Barboza et al. 2013, pg. 262: GOET [GOET003574]; isolectotypes: CORD [CORD00004269], DR [DR054234], US [00027741, acc. # 282274]).

##### Distribution.

Bolivia and Argentina.

##### Notes.

Type material for the varietal names coined by Georg Bitter in the original publication of *S. physalidicalyx* (Bitter 1912) may correspond to duplicates of the type collection of the species itself, making all three names homotypic. In describing var. integrascensBitter (1912) states “var. integrifolia quod in descriptione specie pro typo habui” [var. integrifolia I had in the description of the type of the species] suggesting this name at least is based on *Lorentz & Hieronymus 364*. Bitter often used duplicates with minor leaf variations as material for describing infraspecific variation (Knapp 2013). None of the duplicates of these Lorentz and Hieronymus collections from Salta have annotations in Bitter’s hand, and we have found no other collections of *S. physalidicalyx* made by Lorentz and Hieronymus from “Pasaje de Juramento”.

#### Solanum tweedieanum

Taxon classificationPlantaeSolanalesSolanaceae

Hook., Bot. Mag. 62: tab. 3385. 1835, as “ Tweedianum”

780961FD-3986-5A38-9028-499DB02160AD

Fig. 1D–F

Solanum atriplicifolium Gillies ex Nees, Nov. Act. Acad. Caes. Leop. 19, Suppl. 1: 386. 1843. Type. Argentina. Mendoza: El Diamante, [no date], *J. Gillies s.n.* (lectotype, designated by Barboza et al. 2013, pg. 239): E [E00112916]; isolectotypes: E [E00057545], K[K000585737], NY [00139057]).
Solanum haarupii Bitter, Repert. Spec. Nov. Regni Veg. 11: 210. 1912. Type. Argentina. Mendoza: Estancia Santa Rosa, 1904, *A.C. Jensen-Haarup s.n.* (holotype: UPS; isotype: US [00027594, acc. # 1081085]).
Solanum meizonanthum Bitter, Repert. Spec. Nov. Regni Veg. 11: 214. 1912. Type. Argentina. Entre Ríos: Paraná, 16 Aug 1892, *G. Niederlein 270* (holotype: B [destroyed, F neg. 2783]; lectotype, designated here: F [V0361924F, acc. # 621142]).
Solanum atriplicoides Herter, Rev. Sudamer. Bot. 7: 226. 1943, nom. illeg. superfl. Type. Based on Solanum atriplicifolium Gillies ex Nees

##### Type.

Cultivated at the Glasgow Botanical Garden [protologue] from seeds sent by J. Tweedie from “near Buenos Ayres”, *Anon. s.n.* (lectotype, designated by Edmonds 1972, pg. 102 [as “holotype”], second step designated here: K [K000585739]; isolectotype: K [K000585738]).

##### Distribution.

Bolivia and Argentina.

##### Notes.

Edmonds (1972) stated that the holotype of *S. tweedieanum* was held at K; Barboza et al. (2013) repeated this citation but added reference to a single sheet (K000585739). This is not effective lectotypification under Art. 9.23 of the Code (Turland et al. 2018). The specimen cited as holotype by Barboza et al. (2013) and selected as lectotype here (K000585739) has open flowers and several buds and is a better match for the illustration in the protologue than the other sheet at Kew (K000585738); both specimens are annotated “S. Tweedianum Hook./B^os^. Ayres. Cult.” in W.J. Hooker’s handwriting and bear the herbarium stamp “Herbarium Hookerianum/1867” indicating they come from Hooker’s own herbarium. There is no evidence on the specimens themselves that they were taken from the plants cultivated in Glasgow mentioned in the protologue, nor that they were collected prior to 1835; but the top part of the stem mounted on the sheet we have selected as the lectotype at Kew (K000585739, https://plants.jstor.org/stable/pdf/10.5555/al.ap.specimen.k000585739) is an excellent (mirror-image) match for Tab. 3385 in the protologue (Hooker 1835; see https://www.biodiversitylibrary.org/item/14341#page/36/mode/1up) suggesting it does represent original material. The original, and all subsequent, spelling of the name was “Tweedianum” where John Tweedie’s name was implicitly latinized as “Tweedius” in which the terminal vowel was eliminated. This is not acceptable under Art. 60.9(a)(1) of the Code (Turland et al. 2018) and the name should be formed as “tweedieanum” (e.g., Art. 60.9, Ex. 31, Turland et al. 2018).

### New species descriptions

#### Solanum hunzikeri

Taxon classificationPlantaeSolanalesSolanaceae

Chiarini & Cantero sp. nov.

0288DCCB-7D99-527E-BCF7-1844D3581D44

urn:lsid:ipni.org:names:77212300-1

Figs 1A–C , 2

##### Diagnosis.

Like *Solanum tweedieanum* Hook. but differing in sessile leaves with broadly winged petioles, pedicels in flower longer than 1 cm, larger flowers and anthers more than 1 mm wide.

**Figure 2.**
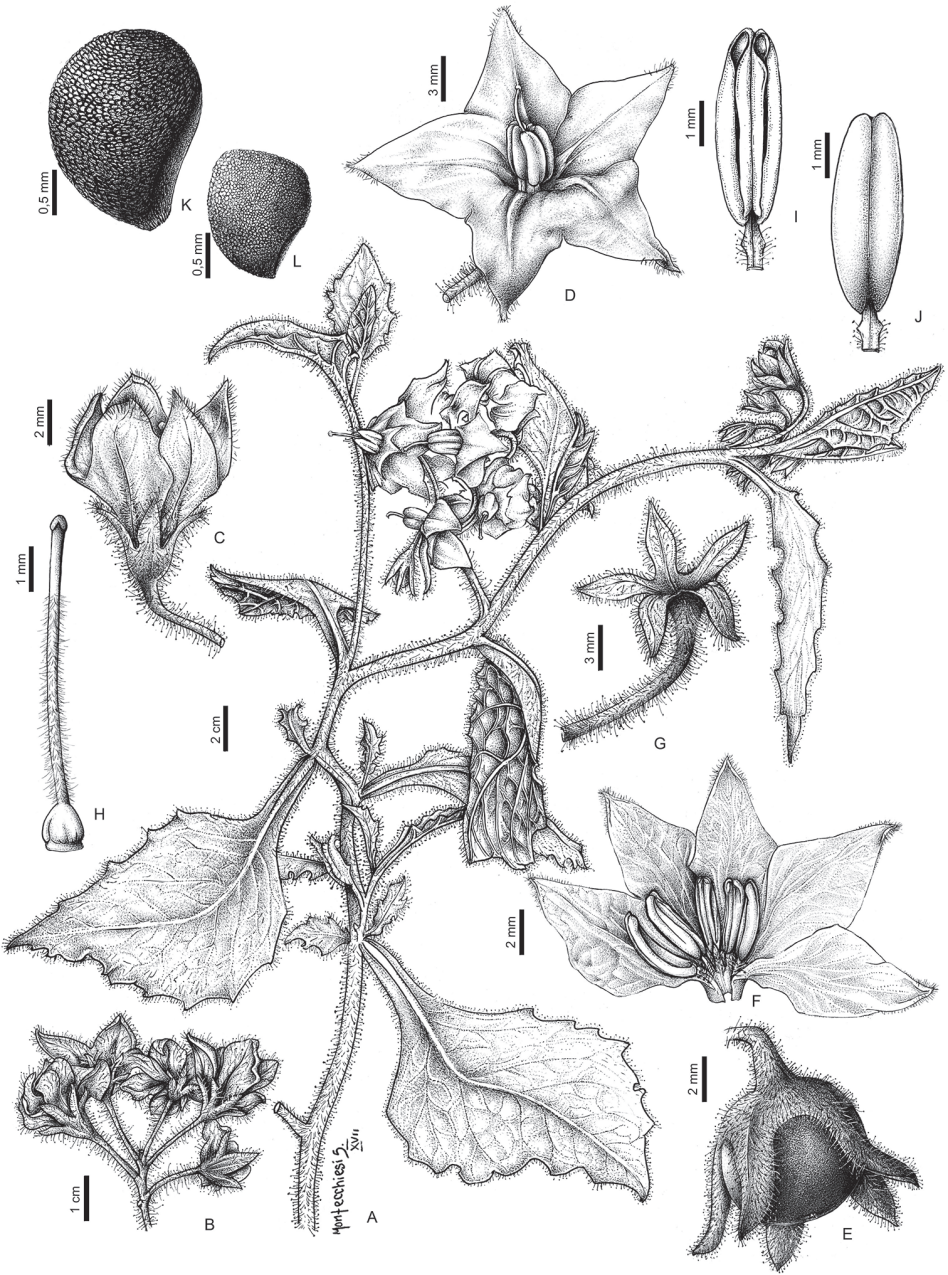
*Solanum hunzikeri* Chiarini & Cantero **A** flowering stem **B** inflorescence **C** flower **D** open flower **E** immature fruit showing the accrescent calyx not completely covering the berry **F** flower showing pubescent adaxial surface of the filaments **G** calyx **H** style with pubescence confined to the portion inside the anther cone **I** adaxial surface of the anther showing the pores elongating with age **J** abaxial surface of the anther **K** seed **L** stone cell (sclereid).

##### Type.

Argentina. Catamarca: Dtto. Ambato, Los Morteritos, Sierra de Ambato, falda E, subiendo desde El Rodeo hacia el Cerro Manchado [Cerro Manchao], 2300–2400 m, 13 Jan 1973, *A.T. Hunziker & R. Subils 22205* (holotype: CORD [CORD00013086]).

##### Description.

Herb or subshrub from a woody base ca. 50 cm tall; stems terete or only slightly angled, densely glandular pubescent with glandular papillae and transparent spreading simple 3–8-celled uniseriate trichomes 0.5–1 mm long, some to 1.5 mm long; bark of older stems pale brown, glabrescent; new growth densely glandular pubescent with simple uniseriate trichomes to 1 mm long. Sympodial units plurifoliate, the leaves not geminate. Leaves simple to shallowly toothed, (2-)4.5–14 cm long, (1.1-)2–7 cm wide, elliptic in outline, membranous or somewhat thick and fleshy, concolorous; adaxial surface moderately and evenly glandular pubescent with transparent spreading, simple uniseriate trichomes ca. 0.5 mm long on the lamina, ca. 1 mm long on the veins; abaxial surface moderately and evenly glandular pubescent like the adaxial surface, but the trichomes denser and longer, to 1.5 mm long; principal veins 4–7 pairs, densely glandular pubescent; base attenuate and strongly decurrent onto the petiole; margins entire or shallowly toothed, the teeth if present 1–2 mm long, 2–3 mm wide, broadly deltate with somewhat rounded tips; apex acute; petioles absent and the leaves sessile or 0–0.1 mm long, the decurrent leaf bases running onto the stem, glandular pubescent like the stems and leaves. Inflorescences 2.5–4 cm long, opposite the leaves, unbranched but occasionally forked (*Rodríguez 1421*), with 10–20 flowers, densely glandular pubescent with transparent spreading simple uniseriate trichomes to 1.5 mm long; peduncle 1.2–2.5 cm long; pedicels 1.3–1.5 cm long, 0.5–0.7 mm in diameter at the base, ca. 1.5 mm in diameter at the apex, spreading at anthesis, densely glandular pubescent, articulated at the base; pedicel scars irregularly spaced 1–2 mm apart. Buds ellipsoid, the corolla ca. halfway exserted from the calyx before anthesis. Flowers 5-merous, perfect. Calyx tube 2–3 mm long, conical, the lobes 2.5–4 mm long, long-triangular, densely glandular pubescent with simple uniseriate trichomes like the pedicels and rest of the inflorescence, the tips acuminate and somewhat recurved at anthesis. Corolla 1.6–2.5 cm in diameter, pale lilac to violet with a yellow-green central star, stellate, lobed ca. 1/2 way to the base, the lobes 5–5.5 mm long, 4–5.5 mm wide, deltate, reflexed or spreading at anthesis, adaxially glabrous, abaxially sparsely glandular papillate especially on the midvein, tips and margins; stamens equal; filament tube 0.35–0.5 mm; free portion of the filaments 1–1.5 mm, almost glabrous, but with a few tangled transparent eglandular simple uniseriate trichomes adaxially; anthers 4–5.5 mm long, 1.25–1.6 mm wide, ellipsoid, yellow, poricidal at the tips, the pores lengthening to slits with age. Ovary conical, glabrous; style 7–8 mm long, densely papillate with a few longer simple trichomes in the lower third; stigma large capitate to slightly bilobed, the surface minutely papillate. Fruit a globose berry, 1–1.2 cm in diameter, green (?) at maturity, opaque, the surface of the pericarp glabrous, thin, matte; fruiting pedicels 1.5–2 cm long, ca. 1.5 mm in diameter at the base, ca. 2 mm in diameter at the apex, somewhat woody, deflexed from the weight of the berry, glandular pubescent to somewhat glabrescent; fruiting calyx accrescent in young fruit tightly investing the berry, the tube 3–5 mm long, later tearing and the berry exposed, the lobes 3–5 mm long, ca. 3 mm wide, appressed to spreading. Seeds ca. 40 per berry, 1.5–2 mm long, 1–1.7 mm wide, flattened teardrop shaped with an apical hilum, reddish brown, the surfaces minutely pitted, testal morphology not clearly seen. Stone cells 10–11 per berry, 1–1.3 mm in diameter, globose, scattered throughout the berry. Chromosome number not known (but see comments on DNA content below).

##### Distribution

**Figure 3.**
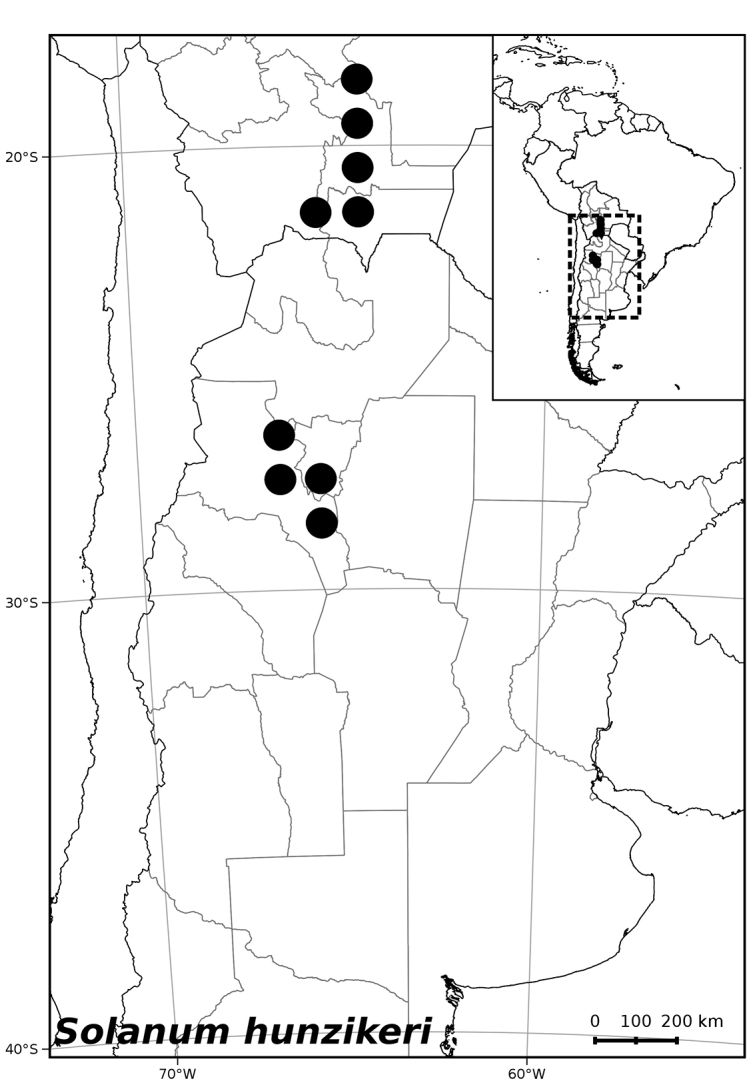
Distribution of *Solanum hunzikeri* Chiarini & Cantero.

(Figure 3). *Solanum hunzikeri* occurs in Argentina in the provinces of Catamarca and adjacent Salta and Tucumán and extends north to Bolivia in the departments of Tarija and Chuquisaca. The distribution is somewhat disjunct possibly due to loss of the wet high elevation foggy grassland habitat in the intervening areas.

##### Ecology and habitat.

*Solanum hunzikeri* is confined to wet cloud forests and foggy grasslands above 1800 m elevation; it also grows in the ecotones between these vegetation types. These foggy grasslands are dominated by tall grasses (e.g., *Festuca hieronymi* Hack., *Cinnagrostis polygama* Griseb., *Elionurus muticus* (Spreng.) Kuntze [Poaceae]) and shrubs (e.g., *Baccharis* spp., *Stevia* spp. [Asteraceae]). *Solanum hunzikeri* can also be locally frequent on open grassy terraces with scattered palms, in narrow valleys with the lower slopes covered in seasonally moist forest dominated by *Parajubaea torallyi* (C.Mart.) Burret (Arecaceae) and with abundant *Podocarpus* spp. (Podocarpaceae) and can be found on steep, stony slopes in undisturbed grassland areas.

##### Etymology.

This species is named in honour of the late Ing. Armando T. Hunziker of IMBIV in Córdoba, whose life work on the Solanaceae inspired a generation of solanologists, in both Argentina and globally.

##### Preliminary conservation status

**(IUCN 2019)**. AOO (80 km^2^ – EN); EOO (97,627 km^2^ – LC). Although the large extent of occurrence would suggest *S. hunzikeri* is not of conservation concern, the limited number of localities, the specialised habitat and the disjunct distribution suggest the species should be considered at risk. *Solanum hunzikeri* occurs in a very restricted habitat in which there are few officially protected areas. In these landscapes the main threat to the ecosystem is over-grazing; the introduction of alien forage species such as *Pennisetum clandestinum* Hochst. ex Chiov. (Poaceae) has severely altered the nature of the high elevation foggy grasslands and forest edges in which *S. hunzikeri* occurs. Although some populations are found in currently protected areas such as the Parque Nacional Aconquija, these areas are considered too small and isolated to provide long term conservation (Brown 1995). Based on the area of occupancy, the number of localities (ca. 8) in a disjunct distribution and threats to the habitat, we assign a preliminary threat status of Vulnerable (VU B2a,biii) for *S. hunzikeri*. The exploration of these relatively inaccessible habitats in the area between the currently known populations of *S. hunzikeri* is a priority.

##### Notes.

*Solanum hunzikeri* had been recognized as distinct from other glandular-pubescent species in Argentina in the early 20^th^ century by the German botanist Georg Bitter as “Solanum catamarcae”, a name already occupied in *Solanum* (*S. catamarcae* Bitter ex Brücher, a synonym of *S. boliviense* Dunal, see Spooner et al. 2019). Morton (1976) in his treatment of *Solanum* for Argentina, cited *Sleumer 2259* as part of his concept of *S. atriplicifolium* and stated “This last appears to be a local form that has the petioles broadly winged nearly throughout instead of at the apex only. According to determinations by Dr. Sleumer this plant was given the unpublished name of “Solanumcatamarcae” by Bitter.” Morton annotated the sheet of *Sleumer 2259* in US as “Solanum atriplicifolium var. sleumeri Morton HOLOTYPE” in 1971 but did not publish the infraspecific epithet; he also annotated *Sleumer 2311* (US) as a paratype of the same.

The species is now known from a wider distribution, and additional specimens have clarified its differences from the widespread and highly variable *S. tweedieanum*. *Solanum hunzikeri* can be distinguished from *S. tweedieanum* populations in similar high elevation areas in its strongly attenuate and winged leaf bases, those of *S. tweedieanum* are more truncate. The single collection we have seen of *S. hunzikeri* with mature fruit (*Rodríguez 1421* from Salta) has the calyx not covering any part of the mature berry; berries of *S. tweedieanum* are tightly covered by the accrescent calyx for at least 50% of their length. More collections of *S. hunzikeri* in fruit are needed to assess these differences. Preliminary data on DNA content for *S. hunzikeri* and *S. tweedieanum* (F. Chiarini unpubl.) show differences but suggest that, like *S. tweedieanum* (Moscone 1992), *S. hunzikeri* is diploid.

##### Additional specimens examined

**(paratypes)**. Argentina. **Catamarca**: Dtto. Ambato, camino desde El Rodeo rumbo al Cerro el Manchado [Manchao], Falda El Morro, 2593 m, 24 Feb 2016, *Barboza et al. 4703* (CORD); Dtto. Ambato, Sierra de Ambato (falda E), subiendo desde El Rodeo hacia el Cerro Manchado [Manchao], 2300 m, 23 Feb 1967, *Hunziker 19073* (CORD, US); Dtto. Pomán, Rumbo al Cerro Manchado [Manchao], Sierra de Ambato, falda E, subiendo El Rincon hacia Las Casitas, rumbo al Cerro Manchado [=Cerro del Manchao], 2300–2500 m, 18 Feb 1970, *Hunziker & Ariza 20319* (CORD); Dtto. Pomán, Rumbo al Cerro Manchado, Sierra de Ambato, falda E, subiendo El Rincon hacia Las Casitas, rumbo al Cerro Manchado [= Cerro del Manchao], 2300–2500 m, 18 Feb 1970, *Hunziker & Ariza 20329* (CORD); Dtto. Ambato, Los Morteritos, Sierra de Ambato, falda E, subiendo desde El Rodeo hacia el Cerro Manchado [Manchao], Los Morteritos, 2300–2400 m, 13 Jan 1973, *Hunziker & Subils 22205* (CORD); Dtto. Ambato, Los Morteritos, Sierra del Ambato, falda E, subiendo desde El Rodeo hacia el Cerro Manchado [Manchao], 2300–2400 m, 13 Jan 1973, *Hunziker & Subils 22206* (CORD); Dtto. Andalgalá, Río Potrero, 2600 m, 13 Feb 1942, *Rohmeder s.n.* (LIL); Dtto. Andalgalá, Río Lampacillo-Río Potrero, Entre Río Lampacillo y Río Potrero, 2700–2900 m, 26 Feb 1951, *Sleumer 1834* (LIL, US); Dtto. Andalgalá, Mesada La Primera, Mesada La Primera, Las Estancias, 1900 m, 11 Feb 1952, *Sleumer 2132* (LIL); Dtto. Andalgalá, Los Queñoales, arriba de la Mesada de Las Rosas, 2300–2400 m, 15 Jan 1952, *Sleumer 2259* (G, LIL, US); Dtto. Andalgalá, Cuesta de la Negrilla, cerca de la Mina de Capillas, 3000–3100 m, 2 Mar 1952, *Sleumer 2690* (CORD, G, US); Dtto. Andalgalá, Cuesta de la Negrilla cerca de la Mina de Capillitas, 3100 m, 2 Mar 1952, *Sleumer 2691* (CORD, G, US, W); Dtto. Andalgalá, Mina de las Capillitas, cerca de los edificios, 2350 m, 2 Jan 1952, *Sleumer 2692* (US, W). **Salta**: Dtto. Cafayate, Peñas Blancas, Cerros de Cajón [Sierras de Quilmes], 4040 m, 30 Mar 1914, *Rodríguez 1421* (BR, CORD, SI). **Tucumán**: Dtto. Alberdi, Escaba, 2300 m, 27 Dec 1913, *Monetti 1838* (LIL); Dtto. Alberdi, Estancia Yunka Suma, Valle del Río Las Chacras [as Catamarca, Dtto. Andalgalá on labels], 1800 m, 23 Feb 1951, *Sleumer 1610* (LIL); Dtto. Alberdi, Cumbres de Suncho, Quebraditas del Portezuelo Sta. Anna [as Catamarca, Dtto. Andalgalá on labels], 2150 m, 8 Feb 1952, *Sleumer 2311* (LIL, US).

Bolivia. **Chuquisaca**: prov. Zudañez, a 82 km de Sucre, entre Tarabuco y Sudanéz, paraje Lambayo, 2756 m, 25 Feb 2004, *Cocucci et al. 3357* (CORD); prov. Azurday, Tarvita, ca. 3 km S of summit on road from Tarvita to Azurduy, 2800 m, 4 Dec 1999, *Wood et al. 15303* (K); prov. Tomina, ca. 1 km W of summit of pass between Villa Tomina and Villa Serrano, 2700 m, 17 Mar 2002, *Wood 17868* (K); prov. Azurduy. Bajando de la cumbre hacia Duraznal en el camino de Azurduy, 2459 m, 11 Dec 2004, *Wood & Huaylla 21130* (K); prov. Tomina, entre Villa Serrano y Tomina, en la cumbre, 2580 m, 4 Mar 2006, *Wood et al. 22394* (K); prov. Zudañez, AMNI El Palmar, AMNI El Palmar, along trail from Torotoro to El Palmar crossing Río Mission Waypu., 2800 m, 2 Feb 2007, *Wood et al. 22612* (K). **Tarija**: Sama, between Tarija and Villazón, 3546 m, 27 Feb 1939, *Balls 6111* (E, K, US); de Tarija a Narváez, 2000–2500 m, 19 Mar 1982, *Kiesling et al. 3734* (SI); de Tarija a Iscayachi, 2000–3000 m, 20 Mar 1982, *Kiesling et al. 3845* (SI); Mun. O’Connor, at the top of the first pass W of Entre Ríos on road to Narváez and Tarija, 1800 m, 21 Jan 2001, *Wood & Goyder 16901* (K).

##### Cultivated.

Argentina. **Córdoba**: IMBIV, Universidad Nacional de Córdoba [plant grown from *Barboza et al. 4703*], 450 m, 15 Feb 2017, *Barboza 4763* (CORD).

#### Solanum marmoratum

Taxon classificationPlantaeSolanalesSolanaceae

Barboza & S. Knapp sp. nov.

4A779109-6CE9-572B-BDF4-99411DEF7009

urn:lsid:ipni.org:names:77212301-1

Figs 4 , 5

##### Diagnosis.

Like *Solanum nitidibaccatum* Bitter but differing in eglandular, white pubescence, strongly winged stems, fleshy calyx lobes that are spreading in fruit and larger berries; also similar to *S. americanum* Mill. but differing in strongly winged stems and dark green mature berries marbled with white markings.

**Figure 4.**
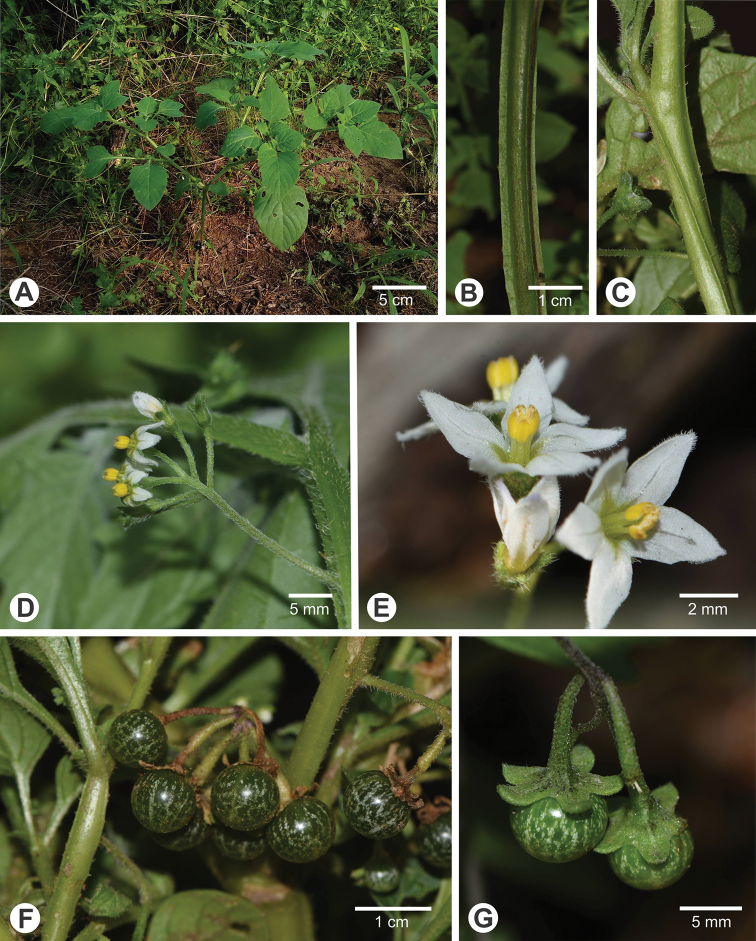
*Solanum marmoratum* Barboza & S. Knapp **A** habit (*Barboza et al. 5099*) **B, C** details of the winged stems (both at the same scale, B from *Barboza et al. 5136*, C from *Barboza et al. 5073*) **D** inflorescence (*Barboza et al. 5136*) **E** flowers, showing the included style and the filaments that elongate with flower age (*Barboza et al. 5136*) **F** mature fruits (*Barboza et al. 5073*) **G** Detail of berries showing the spreading fleshy calyx in fruit (*Barboza et al. 5130*). All photographs by S. Knapp.

##### Type.

Argentina. La Pampa: Dtto. Loventué, 10 km al W de Luan Toro, rumbo a Loventué, 297 m, 9 Feb 2020, *G.E. Barboza, S. Knapp, F. Chiarini & R. Fortunato 5099* (holotype: CORD [CORD00007007]; isotypes: BAB, BM).

**Figure 5.**
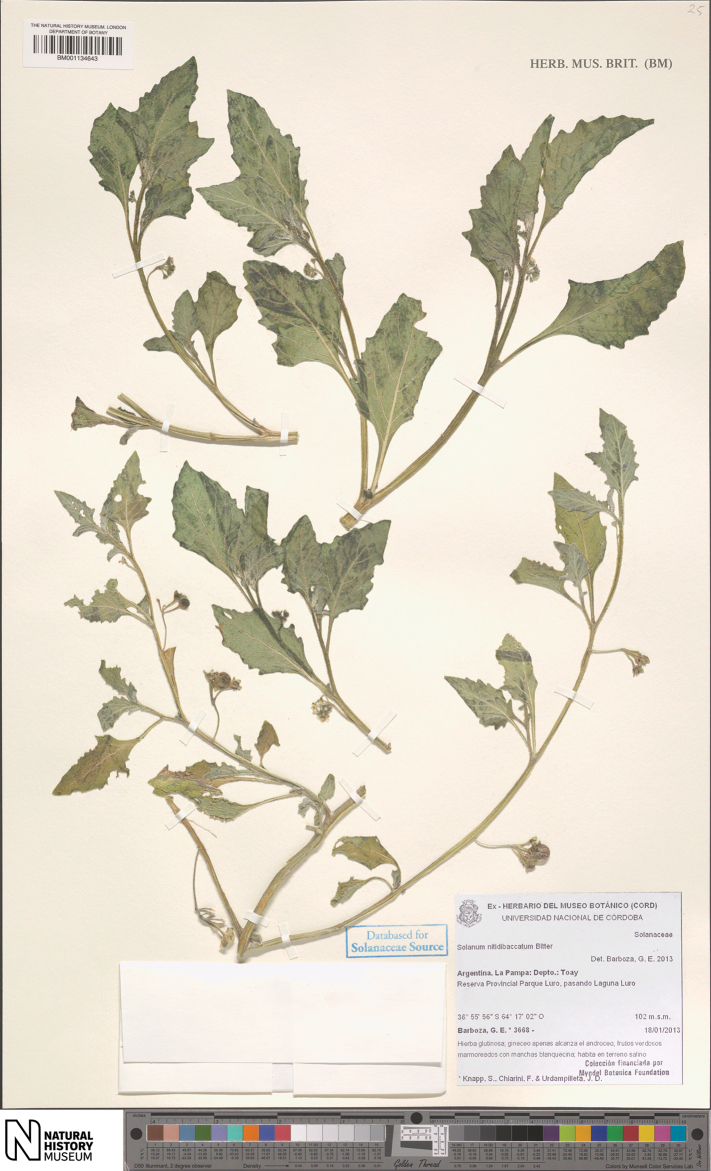
*Solanum marmoratum* Barboza & S. Knapp (*Barboza et al. 3668*, BM [BM001134643]).

##### Description.

Watery annual herb, 10–100 cm tall, sprawling and somewhat prostrate when very large. Stems strongly winged, the wing to 1 mm side, sometimes with spinose processes (old trichome bases), sparsely to moderately pubescent with spreading to appressed eglandular simple 5–8-celled uniseriate trichomes 0.5–1 mm long, these drying white; new growth densely pubescent with eglandular, white simple uniseriate trichomes 0.5–1 mm long; older stems greenish white, not woody. Sympodial units difoliate, the leaves not geminate, axillary shoots common. Leaves simple and shallowly toothed, 2–10 cm long, 1.5–6 cm wide, much larger in older plants, ovate, widest in the lower third, membranous, watery and somewhat succulent, concolorous, very bright green on live plants; adaxial and abaxial surfaces evenly white-pubescent with eglandular simple 5–8-celled uniseriate trichomes 0.5–1 mm long, these longer and denser on the veins; principal veins 5–6 pairs; base attenuate onto the petiole; margins shallowly and irregularly toothed, the teeth 2–4 mm long, 2.4- mm wide, broadly deltate, with blunt tips; apex acute; petioles 0.5–2.5 cm long, somewhat winged from the attenuate leaf base, pubescent with simple uniseriate trichomes like the stems and leaves. Inflorescences (1)2–3 cm long, internodal and extraaxillary, unbranched, with 5–7 flowers clustered at the tip, usually only 1–2 open at a time, sparsely and evenly pubescent with antrorse simple uniseriate trichomes 0.5–1 mm long like the stems and leaves; peduncle 1.4–2.5 cm long; pedicels 0.4 cm long, ca. 0.5 mm in diameter at the base, ca. 0.6 mm in diameter at the apex, slightly tapering, spreading, eglandular pubescent like the rest of the inflorescence, articulated at the base; pedicel scars tightly packed at the tip of the inflorescence, 0.5–1.5 mm apart. Buds broadly ellipsoid, the corolla included in the calyx tube until just before anthesis. Flowers 5-merous, perfect. Calyx tube 1.2–1.5 mm long, cup-shaped, the lobes 1–1.5 mm, narrowly deltate-triangular, fleshy and recurved in live plants, sparsely pubescent with eglandular white trichomes on both surfaces like the rest of the plant. Corolla 0.5–0.8 cm in diameter, white with a green central star, stellate, lobed ca. halfway to the base, the lobes ca. 2.5 mm long, ca. 2 mm wide, spreading to slightly reflexed at anthesis (flowers closing daily and lasting for several days), adaxially glabrous, abaxially densely pubescent with tiny simple uniseriate trichomes especially at the tips. Stamens equal or slightly unequal with one anther marginally longer than the rest; filament tube ca. 0.1 mm long; free portion of the filaments 0.5–1 mm long, elongating through anthesis, with a few tangled transparent simple uniseriate trichomes adaxially; anthers 1–1.5 mm long 0.6–1 mm wide, ellipsoid, yellow, poricidal at the tips, the pores elongating with age. Ovary conical, glabrous; style 2–2.5 mm, included within the anther cone or the stigma just beyond, densely papillate in the lower 3/4; stigma large capitate, held at the level of the anthers when flowers first open, later included within the anther cone, bright green in life plants, the surfaces minutely papillate. Fruit a globose berry, 0.8–1.5 cm in diameter, dark green marbled with white at maturity, glabrous, translucent, the pericarp surface thin, shiny; fruiting pedicels 1.2–1.5 cm long, ca. 1 mm in diameter at the base, ca. 1.5 mm in diameter at the apex, fleshy and watery, tapering to the spreading calyx, strongly deflexed at maturity, with a distinct bend at the pedicel base; fruiting calyx somewhat expanded, the tube 3–4 mm long, the lobes 4–5 mm long, ca. 3 mm wide, spreading and fleshy, the tips rounded. Seeds 50–70 per berry, ca. 2 mm long, ca. 1.7 mm wide, flattened teardrop shape with an apical hilum, pale tan to reddish brown, the surfaces minutely pitted, the testal cells mostly rectangular to pentagonal in outline, more sinuate towards the seed centre. Stone cells 1–2, 1–1.1 mm in diameter, found randomly positioned in the berry. Chromosome number: not known.

##### Distribution

**Figure 6.**
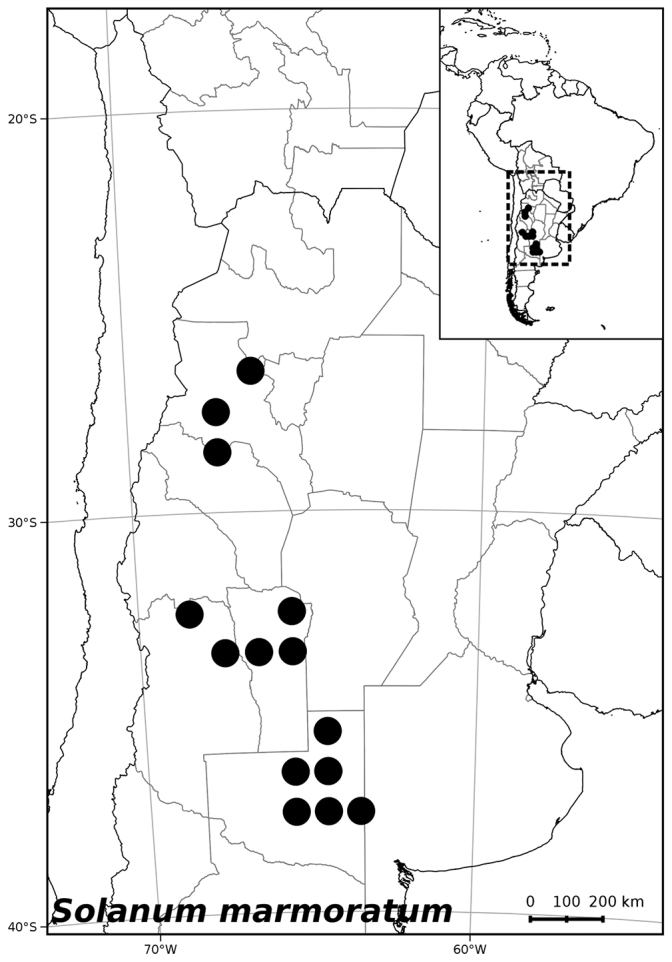
Distribution of *Solanum marmoratum* Barboza & S. Knapp.

(Figure 6). *Solanum marmoratum* is endemic to Argentina and occurs in the provinces of Catamarca, La Pampa, La Rioja, San Juan and San Luis; we expect it also to be found in Mendoza, because several collections are known from Desaguadero (San Luis) a locality very close to the provincial border that crosses through uniform habitat.

##### Ecology and habitat.

*Solanum marmoratum* is found in shady areas in *Prosopis* woodlands (Fig. 4A) and at the edges of arable fields; it usually grows under trees and shrubs with a number of other herbaceous plants such as *S. tweedieanum*, various species of Asteraceae and grasses. Specimens have been collected from 200 to 1400 m elevation.

##### Etymology.

The species is named for its distinctive marbled berries (Fig. 4F, G) that easily distinguish it from the similar tiny-flowered eglandular species *S. americanum*.

##### Preliminary conservation status

**(IUCN 2019)**. AOO (84 km^2^ – EN); EOO (239,336 km^2^ – LC). *Solanum marmoratum* is a relatively widespread species, the extent of occurrence suggests is should be given a status of least concern. The small area of occupancy perhaps reflects a lack of collecting in the dry forest and partially degraded habitats where *S. marmoratum* occurs. The number of localities (ca. 9) is probably an underestimate due to the widespread perception that these habitats are not interesting; most collections are quite old and the species has not be collected recently (except by us). The large-scale conversion of land in the range of *S. marmoratum* to intensive monoculture of commercial crops such as maize, peanuts and sunflowers poses a risk for this and other species in these habitats; use of herbicides and elimination of patches of forest leave little room for even weedy species to persist. We suggest a preliminary threat status of Least Concern (LC) to *S. marmoratum*, but the widespread habitat conversion in central Argentina warrants further studies as to population status across the species’ historical range.

##### Notes.

*Solanum marmoratum* has long confused botanists working with Argentinian solanums. In the herbarium at CORD specimens of *S. marmoratum* collected by P. Steibel in the province of San Luis were the subject of correspondence with A.T. Hunziker over their identification; they were tentatively identified as *S. adventitium* Polg., a synonym of *S. americanum* described from adventive material in Hungary (Särkinen et al. 2018). None of the pre-1970s specimens we have seen were cited in Morton (1976), but *Semper s.n.* at US (barcode 02837698) was annotated “Solanum dolichopteryx Morton, paratype” by C.V. Morton in 1971. We have not found specimens annotated as other types at US or elsewhere. A.T. Hunziker had kept specimens of this species aside with the herbarium name “Solanum alatocaule”, a reference to the strongly winged stems (Fig. 4B, C) on the folder, but never described it. We collected *S. marmoratum* in 2013 (*Barboza et al. 3668*) along with *S. tweedieanum*, and mistakenly noted the leaves of *S. marmoratum* as sticky (see Fig. 5); it was only examination of the dried specimens that alerted us to our error. Careful examination of all morelloid collections at CORD in early 2020 showed the distinctness of *S. marmoratum*, and its relatively widespread distribution.

The flowers of *S. marmoratum* are among the tiniest in the morelloid solanums (Fig. 4D, E) rivalled only by the globally distributed *S. americanum* and *S. nitidibaccatum* Bitter and the North American *S. emulans* Raf. (see Knapp et al. 2019). *Solanum nitidibaccatum* also has somewhat marbled berries but is always extremely sticky and covered with glandular trichomes, in contrast to the eglandular pubescence of *S. marmoratum*. *Solanum americanum* and *S. emulans* both have eglandular pubescence but have purplish black rather than green marbled berries. The fleshy spreading calyx lobes of *S. marmoratum* (Fig. 4G) are distinct from those of all of these taxa with tiny flowers.

*Solanum marmoratum* appears to be highly autogamous and is perhaps entirely self-fertilising. The style is completely included within the anther cone (Fig. 4D, E) and the filaments appear to elongate through anthesis (see. Fig. 4E) bringing the style further into the cone as the flower ages. Flowers stay open for several days (closing at night) and in cultivation the plant goes from bud to flower to fruit in 15–18 days with all flowers setting fruit. Over the course of anthesis the style becomes enclosed in the anther cone (Fig. 4E), with the anthers as they dehisce leaving pollen directly on the stigma. Ripe berries last more than two weeks after being gathered from desiccated plants, remaining unchanged as to colour or odour.

##### Additional specimens examined

**(paratypes)**. Argentina. **Catamarca**: Dtto. Santa María, Chiñucán, Sierra de la Aconquija, falda O, Chiñucán, 12 Apr 1948, *Reales 1264* (CORD); Dtto. Belén, Yacutula, Mar 1879, *Schickendantz 113* (CORD). **La Pampa**: Dtto. Toay, Reserva Provincial Parque Luro, pasando la laguna Luro, 233 m, 18 Jan 2013, *Barboza et al. 3668* (BM, CORD, SI); Dtto. Utracán, Valle de Daza, rumbo a la Laguna El Loro, 10–12 km de la RP 18, 290 m, 8 Feb 2020, *Barboza et al. 5073* (BM, CORD); Dtto. Toay, Reserva Parque Luro, ingreso S por ruta 35 desde General Acha, 116 m, 9 Feb 2020, *Barboza et al. 5079* (BM, CORD); Bajo Lucero, cruce entre RP 11 y RP 10, 255 m, 9 Feb 2020, *Barboza et al. 5094* (BM, CORD); Dtto. Atreucó, sin. loc., Mar 1960, *Cano 960* (US); Dtto. Rancul, Chamaicó, 2 Mar 1984, *Steibel & Troiani 7960* (CORD); Chamaicó, 2 Mar 1984, *Steibel & Troiani 7963* (CORD); Dtto. Atreucó, Laguna Chillhué, 5 Apr 1984, *Steibel et al. 8035* (CORD); Dtto. Capital, Barrancas Coloradas, 28 Feb 1991, *Steibel 10111* (CORD); Dtto. Toay, Parque Luro, 8 Mar 1991, *Steibel 10118* (CORD); Dtto. Capital, El Guanaco, 30 km al N de Santa Rosa, 13 Feb 1977, *Troiani 4688* (CORD); Dtto. Atreucó, Laguna Chillhué, 13 Mar 1982, *Troiani et al. 6820* (CORD); Dtto. Loventué, Luan Toro, 10 km al W, 10 Feb 1985, *Troiani 8564* (CORD). **La Rioja**: Dtto. Famatina, Ruta 40 [now Ruta Prov. 11] (km 640/641), yendo de Famatina a Tinogasta, 20 Mar 1960, *Hunziker et al. 15172* (CORD); Ruta 40 [now Ruta Prov. 11] (km 692), yendo de Famatina a Tinogasta, entre Santa Cruz y el límite con Catamarca, 20 Mar 1960, *Hunziker et al. 15206* (CORD,US). **San Juan**: Dtto. Sarmiento, a 2 km de la estancia El Acequión, desde El Pedernal hacia el enpalme con Ruta provincial 412, por Ruta Prov. 312, 1400 m, 9 Apr 2004, *Matesevach 10 C*, (CORD). **San Luis**: Dtto. General Pedernera, Villa Mercedes, Estancia Agropecuaria INTA San Luis, lote 16, bajo, 510 m, 9 Jan 1969, *Anderson & Galvani 1511* (CORD); Sierra El Morro, cuenca interior, querencia pisoteada, 1200 m, 27 Dec 1977, *Anderson et al. 3427* (CORD); Dtto. Capital, Estancia Las Tres Marías, 30 km al sur de San Luis-Quemado, 700 m, 21 Mar 1979, *Anderson et al. 3643* (CORD); Dtto. Chacabuco, Concarán, cerca de 2 km desde Concarán rumbo a Santa Rosa de Conlara, por el camino de tierra (RP 23), 660 m, 24 Feb 2020, *Barboza et al. 5130* (BM, CORD); Concarán, cerca de 5.5 km desde Concarán rumbo a Santa Rosa de Conlara, por el camino de tierra (RP 23), 652 m, 24 Feb 2020, *Barboza et al. 5136* (BM, CORD); Dtto. Capital, Potrero de los Funes, 2 Apr 1989, *Del Vitto & Petenatti 3455* (CORD); Los Puquios, a 200 m del badén sobre el río Los Puquios en la ruta El Volcán-Cruz de Piedra, rumbo a Cruz de Piedra, 21 May 1972, *Giordano & Guerreiro 23* (CORD); Dtto. Chacabuco, a ca. 3 km al N de Concarán, rumbo a Santa Rosa, por el viejo camino de tierra, 17 Feb 1989, *Hunziker et al. 25335* (CORD); Dtto. Belgrano, Sierra del Gigante (falda O), Desaguadero, inmediaciones de Paso de Tropas, 7 Apr 1944, *Ruiz Leal 9191* (CORD); Dtto. Capital, entre Estación Jarilla y Desaguadero., 500 m, 7 Apr 1944, *Semper s.n.* (BM, US).

#### Solanum tiinae

Taxon classificationPlantaeSolanalesSolanaceae

Barboza & S. Knapp sp. nov.

D607EEA4-CFDD-523F-83FA-D0EA2C586D25

urn:lsid:ipni.org:names:77212302-1

Figs 7 , 8

##### Diagnosis.

Like *Solanum aloysiifolium* Dunal but differing in narrower leaves decurrent onto the stems, antrorse pubescence, ellipsoid buds and strongly deflexed fruiting pedicels.

**Figure 7.**
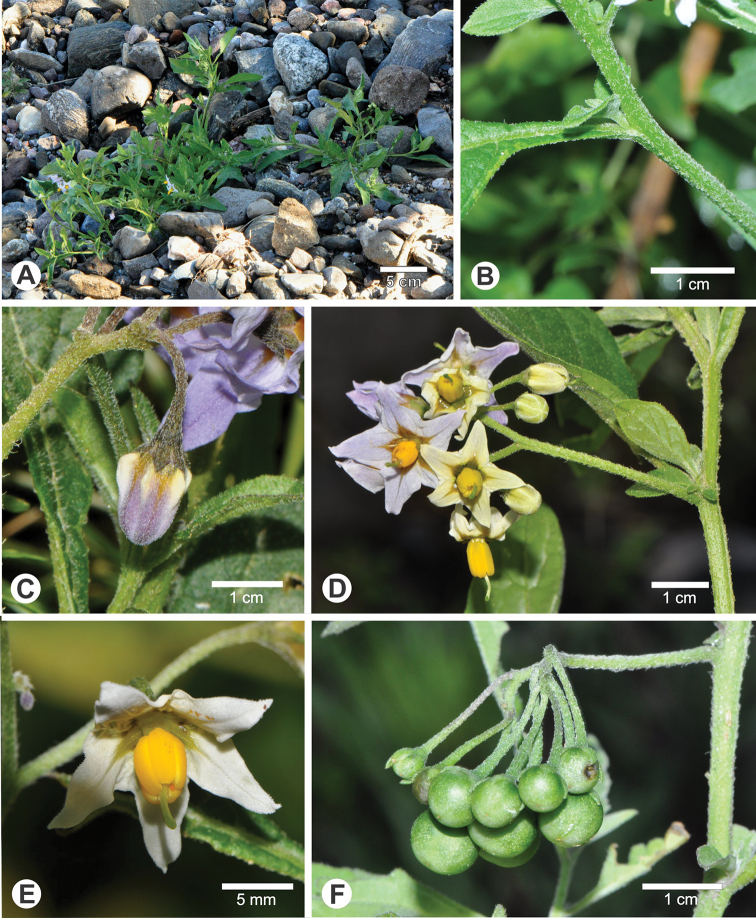
*Solanum tiinae* Barboza & S. Knapp **A** habit (*Barboza et al. 3491*) **B** details of the attenuate leaf base and winged stems with antrorse trichomes (*Barboza et al. 3491*) **C** flower bud (*Barboza et al. 3496*) **D** inflorescence (*Barboza et al. 3491*) **E** flowers at anthesis, note the changing colour and size (*Barboza et al. 3491*) **F** mature fruits (*Barboza et al. 3491*). All photographs by S. Knapp.

##### Type.

Argentina. Tucumán: Dtto. Tafí del Valle, El Infiernillo, en el parador, 3042 m, 13 Feb 2012, *G.E. Barboza*, *S. Knapp & T. Särkinen 3496* (holotype: CORD [CORD00013848]; isotypes: BM [BM001115408, BM001115409], others to be distributed).

**Figure 8.**
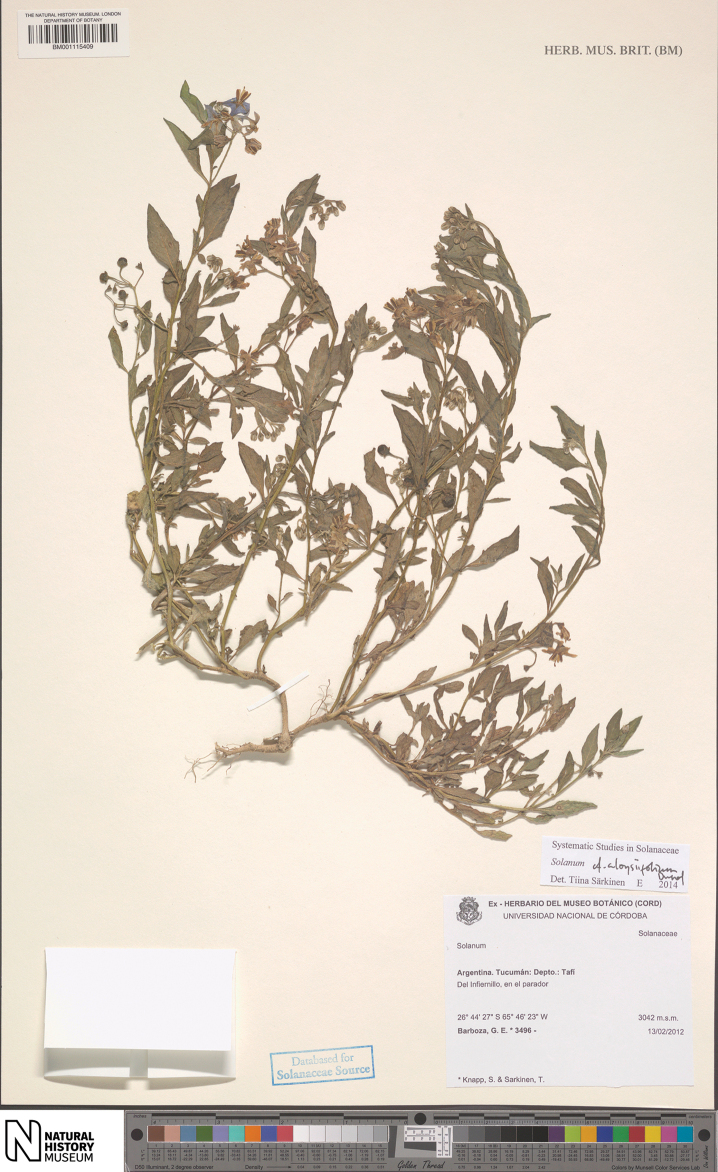
*Solanum tiinae* Barboza & S. Knapp (isotype: *Barboza et al. 3496*, BM [BM001115409]).

##### Description.

Perennial herbs or subshrubs sprawling from a woody base, to 50 cm tall. Stems narrowly winged, the wing to 0.5 mm wide, often invested with spinose processes (enlarged trichome bases), sparsely pubescent with antrorse eglandular, simple uniseriate trichomes, 6–10-celled, ca. 0.5 mm long, these white when dry; new growth densely to moderately pubescent with antrorse eglandular, simple 2–8-celled uniseriate trichomes, ca. 0.5 mm long; bark of older stems pale greenish brown, glabrescent. Sympodial units plurifoliate, the leaves not geminate. Leaves simple, 2–5 cm long, 0.6–2 cm wide, narrowly elliptic to almost lanceolate in some individuals, membranous, concolorous; adaxial surfaces sparsely and evenly pubescent with antrorse eglandular simple 2–4-celled uniseriate trichomes to 0.5 mm long, the trichomes slightly longer on the veins, white when dry; abaxial surfaces with similar, but denser eglandular antrorse pubescence; principal veins 4–6 pairs, drying yellow, especially abaxially; base attenuate and decurrent onto the winged stem and the leaves sessile or nearly so; margins entire or with a few teeth ca. 2 mm long, ca. 2 mm wide with blunt tips in the lower third to half; apex acute to slightly blunt-tipped; petiole absent to 0.2 mm long, eglandular pubescent like the stems and leaves. Inflorescences 2.5–5 cm long, opposite the leaves or internodal, forked with 2 short branches, with 10–20 flowers clustered at the tips of the inflorescence branches, sparsely pubescent with antrorse eglandular simple uniseriate trichomes like those of the stems; peduncle 1.2–2.5 cm long; pedicels 0.8–1 cm long, ca. 0.5 mm in diameter at the base, ca. 1 mm in diameter at the apex, strongly tapering, spreading to somewhat deflexed at anthesis, sparsely to moderately sparsely pubescent with antrorse eglandular simple uniseriate trichomes like the rest of the inflorescence, articulated at the base; pedicel scars clustered at the tips of the inflorescence branches, ca. 0.5 mm apart. Buds ellipsoid to somewhat turbinate (widest in lower third), the corolla strongly exserted from the calyx tube before anthesis, the style sometimes exserted from the bud before anthesis. Flowers 5-merous, perfect. Calyx tube 1.5–2 mm long, conical, the lobes (0.5)1–2 mm long, deltate with lanceolate tips, the sinuses rounded, sparsely pubescent with antrorse eglandular trichomes like the pedicels. Corolla 1.2–2.2 cm in diameter, white, pale violet or white tinged with violet, sometimes changing colour through anthesis, with a brownish yellow to yellow-green central star edged with brownish purple, stellate, lobed halfway to the base, the lobes 5–8 mm long, 4–5 mm wide, deltate to triangular, spreading or slightly reflexed at anthesis, adaxially glabrous, abaxially densely pubescent with eglandular papillae and simple uniseriate trichomes to 0.2 mm long. Stamens equal; filament tube minute; free portion of the filaments 0.5–1 mm long, adaxially densely pubescent with tangled transparent simple uniseriate trichomes; anthers 4–5 mm long, 1–1.25 mm wide, ellipsoid, yellow, the abaxial surfaces occasionally papillate, poricidal at the tips, the pores lengthening to slits with age. Ovary conical, glabrous; style 7–10 mm long, pubescent along almost the entire length, more densely in the lower half with tangled transparent simple trichomes to 0.5 mm long; stigma capitate to clavate, bright green in live plants, the surface minutely papillose. Fruit a globose berry, 0.8–0.9 cm in diameter, green with tiny white spots (immature?), opaque, the pericarp surface thin, matte, glabrous; fruiting pedicels 0.8–1 cm long, ca. 0.75 mm in diameter at the base, ca. 1.5 mm in diameter at the apex, thickened but not woody, strongly deflexed with a distinct bend at the pedicel base; fruiting calyx not enlarged or accrescent, the lobes appressed to the surface of the berry. Seeds 10–30 per berry, 1.7–2 mm long, 1–1.5 mm wide, not markedly flattened, teardrop shaped with an apical hilum, pale tan, the surfaces minutely pitted, the testal cells sinuate in outline. Stone cells 4–9 per berry, 0.7–1.5 mm in diameter, 2 usually larger than the rest. Chromosome number: n=12 (Moscone 1992, as S. lorentzii Bitter var. montigenum C.V.Morton).

##### Distribution

**Figure 9.**
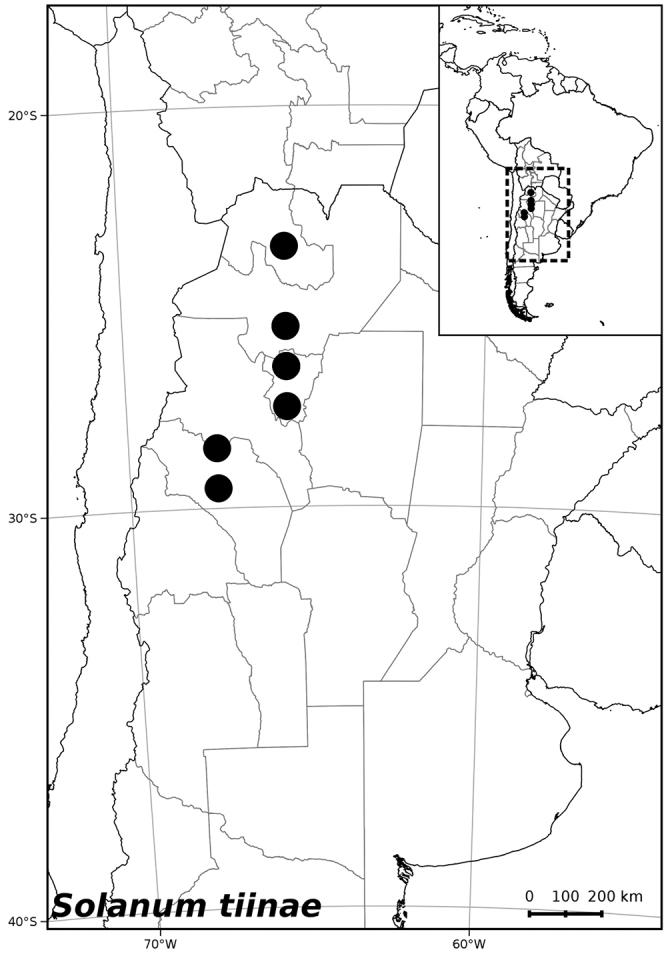
Distribution of *Solanum tiinae* Barboza & S. Knapp.

(Figure 9). *Solanum tiinae* is endemic to Argentina; it has been collected from the provinces of Jujuy, La Rioja, Salta and Tucumán, with most collections from the area around the type locality at El Infiernillo.

##### Ecology and habitat.

*Solanum tiinae* grows among rocks and in open areas in pre-puna habitats in the Andes (Fig. 7A), from 2400 to 4000 m elevation.

##### Etymology.

*Solanum tiinae* is named in honour of our long-term collaborator and colleague Dr. Tiina Särkinen of the Royal Botanic Garden Edinburgh; she was the first to notice the uniqueness of these plants, giving them the field name “Solanum misterioso” while in the field in 2012.

##### Preliminary conservation status

**(IUCN 2019)**. AOO (76 km^2^ – EN); EOO (41,143 km^2^ – NT). Most collections of *S. tiinae* are from a very few commonly visited localities and the main road between Tafí del Valle and Amaicha del Valle in the province of Tucumán. *Solanum tiinae* is not found in protected areas, and based on the number of localities (ca. 5), the area of occupancy and the extent of occurrence, we assign a preliminary threat status of Vulnerable (VU B2a,biii). Where it occurs *S. tiinae* is not common or weedy, although it does grow in open areas.

##### Notes.

It is surprising that *S. tiinae* has not been described previously, as the area from which the type and many other collections come is one of the most intensively collected Andean areas in Argentina. None of the collections we cite here were cited in Morton (1976). We have encountered specimens of *S. tiinae* identified as *S. aloysiifolium* (and its synonyms, see Barboza et al. 2013) and *S. cochabambense*. It is similar to those taxa in its forked inflorescence with a long peduncle, but differs from *S. aloysiifolium* in its larger, less deeply stellate purple or purplish cream (rather than white) corollas, and from *S. cochabambense* in its smaller habit and winged stems. The strongly antrorse pubescence of *S. tiinae* is distinctive and not found in either *S. aloysiifolium* or *S. cochabambense*.

*Solanum tiinae* also resembles the highly variable species *S. salicifolium*, from which it can be distinguished by its shorter (1–2 mm versus 2.5–3 mm long) calyx lobes, the strongly antrorse pubescence (Fig. 7B), the strictly furcate (versus only occasionally once branched) inflorescences with more flowers (10–20 versus 4–10) (Fig. 7B, C) and the calyx lobes (Fig. 7F) that are tightly appressed to the berry (versus spreading and slightly recurved). These two taxa have been collected in the same habitat (e.g., *Barboza et al. 3491*, *S. tiinae* and *Barboza et al. 3494*, *S. salicifolium* from km 92 on the Amaicha del Valle to Tafí del Valle road) and can be easily distinguished in the field using corolla shape – those of *S. salicifolium* are deeply stellate with relatively narrow lobes, while those of *S. tiinae* are less deeply and more broadly lobed (Fig. 7E).

##### Additional specimens examined

**(paratypes)**. Argentina. **Jujuy**: Dtto. Tilcara, Sierra de Zenta, 4000 m, Feb 1931, *Budin 7471* (CORD). **La Rioja**: Dtto. Famatina, Rodeo de las Vacas, 3000–4000 m, Feb 1913, *Flossdorf 55* (SI); Quebrada Encrucijada, 3500–5000 m, Mar 1913, *Flossdorf 56* (SI). **Salta**: Dtto. San Carlos, Amblayo, 2371 m, 16 Mar 1943, *Hunziker 2623* (CORD); Dtto. Cachi, Ruta Prov. 33, de Piedra del Molino a El Carril, La Herradura, 3110 m, 26 Feb 2009, *Zuloaga et al. 11256* (CORD, SI). **Tucumán**: Dtto. Tafí del Valle, Pinar de los Ciervos, Km 70, 2400 m, 6 Mar 1998, *Barboza et al. 139* (CORD); Dtto. Tafí del Valle, Pinar de Los Ciervos, km 70, 2400 m, 6 Mar 1998, *Barboza et al. 140* (CORD); Dtto. Tafí del Valle, entre Tafì del Valle y Amaichá: Km 76, 6 Mar 1998, *Barboza et al. 150* (CORD); Dtto. Tafí del Valle, entre Tafì del Valle y Amaichá: Km 76, 6 Mar 1998, *Barboza et al. 151* (CORD); Dtto. Tafí del Valle, entre Tafí del Valle y Amaicha, 6 Mar 1998, *Barboza et al. 152* (CORD); Dtto. Tafí del Valle, El Infiernillo, 2920 m, 19 Mar 2006, *Barboza et al. 1705* (CORD); Dtto. Tafí del Valle, Carapunco, rumbo a Amaicha del Valle por RP307, 2864 m, 24 Feb 2009, *Barboza et al. 2167* (CORD); El Infiernillo, 2960 m, 24 Feb 2009, *Barboza et al. 2172* (CORD); Dtto. Tafí del Valle, La Quebradita, a unos pocos km de Tafí del valle rumbo a Amaicha del Valle, 2053 m, 21 Feb 2011, *Barboza et al. 3014* (CORD); Dtto. Tafí del Valle, a 28 km de Tafí rumbo a Amaicha del Valle, 2857 m, 21 Feb 2011, *Barboza et al. 3019* (CORD); Dtto. Tafí del Valle, desde Amaicha del Valle rumbo a Tafí del Valle, entre km 92–91, 3000 m, 13 Feb 2012, *Barboza et al. 3491* (BM, CORD); Dtto. Tafí del Valle, entre Tafì del Valle y Amaichá, El Infiernillo, 19 Feb 1988, *Cocucci et al. 293* (CORD); Tafí del Valle, 2500 m, 24 Feb 1998, *Cocucci 989* (CORD); Dtto. Tafí del Valle, Km. 82, al N de Tafí del Valle, hacia la quebrada del Barón, 3100 m, 26 Feb 1959, *Diers 285* (SI); Dtto. Tafí del Valle, El Molle, en el camino entre Tafí del Valle y Amaicha, km 91–92, 2800–2900 m, 12 Feb 1986, *Hunziker et al. 24878* (BM, CORD [x2], E, MO); Dtto. Tafí del Valle, El Molle, en el camino entre Tafí del Valle y Amaicha, km 91–92, 2800–2900 m, 12 Feb 1986, *Hunziker et al. 24879* (CORD); Dtto. Tafí del Valle, viniendo desde Tafí del Valle, rumbo a Amaicha del Valle, entre kms 75 y 76, 2600- 2700 m, 14 Dec 1995, *Hunziker et al. 25546* (CORD); Dtto. Tafí del Valle, El Infiernillo, 22 km de Tafí del Valle, 2950 m, 18 Mar 1972, *Krapovickas et al. 21885* (CTES); Tafí, 2000 m, 4 Dec 1908, *Lillo 8691* (CORD, LIL, SI, US); Dtto. Tafí del Valle, La Ciénaga; Sierra de Tucuman, 10 Jan 1874, *Lorentz & Niederlein 565* (CORD); Dtto. Tafí del Valle, 16 km N de Tafí del Valle, Mojon, K [km] 78, 16 Mar 1972, *Maruñak et al.209* (CTES); Infiernillo, Tafí del Valle, 1850 m, 2 Mar 1972, *Meyer s.n.* (LIL); Dtto. Tafí del Valle, Ruta 40, ca. 10 km NW de Tafí del Valle, Cumbres Calchaquíes, 2985 m, 26 Jan 2007, *Paula-Souza et al. 7912* (CTES); Dtto. Tafí del Valle, Infiernillo, 3040 m, 8 Mar 1955, *de la Sota 236* (CORD); Dtto. Tafí del Valle, Cumbres Calchaquí, Quebrada Honda, 3100 m, 23 Jan 1952, *Sparre 9232* (CORD); Dtto. Tafí del Valle, Cumbres Calchaquí, Quebrada Honda, 3100 m, 23 Jan 1952, *Sparre 9233* (CORD); Dtto. Tafí del Valle, Tafi del Valle, 4 Dec 1960, *Subils & Articó 285* (BM, CORD); Dtto. Tafí del Valle, Colalao del Valle, alrededores, 17 Feb 1979, *Subils & Bernardello 2670* (CORD); Dtto. Tafí del Valle, pasando Tafí del Valle rumbo a Amaicha, Carapunco, 2942 m, 1 Apr 2012, *Urdampilleta et al. 760* (CORD); Dtto. Tafí del Valle, Tafí del Valle, 2900 m, 29 Jul 1971, *Without Collector s.n.* (BAA); Dtto. Tafí del Valle, El Infiernillo, RP 307, 2995 m, 18 Mar 2018, *Zuloaga et al. 16415* (SI).

### Artificial key to morelloid species occurring in Argentina

**Table:** 

1	Plants glandular pubescent, sticky to the touch; glandular trichomes usually several-celled	**2**
–	Plants not glandular pubescent or sticky to the touch; glandular trichomes, if present, very small and usually papillate	**14**
2	Corolla campanulate, purplish blue; anthers with the connective enlarged abaxially	**3**
–	Corolla variously stellate, white or purple; anthers without obvious connective enlargement	**4**
3	Inflorescence forked or several times branched, with 11–50+ flowers; calyx lobes triangular, shorter than the tube; fruiting calyx scarcely accrescent	***Solanum fiebrigii* Bitter**
–	Inflorescence unbranched, subumbellate, with 4–7 flowers; calyx lobes narrowly triangular, longer than the tube; fruiting calyx accrescent, but leaving the berry exposed	***Solanum sinuatiexcisum* Bitter**
4	Anthers 0.8–2.5 mm long	**5**
–	Anthers 2.5–5(6) mm long	**8**
5	Calyx lobes broadly deltate with rounded tips	***Solanum grandidentatum* Phil.**
–	Calyx lobes variously triangular with pointed tips	**6**
6	Calyx completely enclosing the bud; fruiting calyx covering more than half the berry; mature berry green; inflorescence leaf-opposed; plants delicate annuals	***Solanum sarrachoides* Sendtn.**
–	Calyx not completely enclosing the bud; fruiting calyx covering less than half the berry; mature berry green with white marbling; inflorescence usually internodal, occasionally some inflorescences on a plant almost leaf-opposed; plants woody at the base, or more robust annual weeds	**7**
7	Anthers ca. 2 mm long; fruiting calyx lobes spreading, with very marked venation; plants woody at the base	***Solanum physalifolium* Rusby**
–	Anthers less than 1 mm long; fruiting calyx lobes not markedly spreading, the venation not marked; plants usually not woody at the base	***Solanum nitidibaccatum* Bitter**
8	Erect herbs or small shrubs, usually woody at the base; buds elongate ellipsoid, the corolla strongly exserted from the calyx in bud; inflorescence furcate (rarely unbranched); berry purple or green, less than 0.6 cm in diameter	***Solanum glandulosipilosum* Bitter**
–	Decumbent or spreading herbs, sometimes woody at the base; buds broadly ellipsoid, variously covered by the calyx in bud; inflorescence unbranched (rarely furcate); berry green or green marbled with white, usually more than 0.6 cm in diameter	**9**
9	Fruiting calyx accrescent and inflated, completely enclosing the berry	***Solanum physalidicalyx* Bitter**
–	Fruiting calyx variously accrescent, but never inflated, only partially enclosing the berry if at all	**10**
10	Fruiting calyx lobes spreading to reflexed, not appressed to the basal portion of the berry; stone cells absent in berry	**11**
–	Fruiting calyx lobes accrescent, appressed to the berry at least in early fruit, not spreading; stone cells present or absent in berry	**12**
11	Anthers 3–3.8 mm long, wider at the base; corolla strongly exserted from the bud before anthesis, exceeding the tips of the lobes	***Solanum woodii* Särkinen & S. Knapp**
–	Anthers 2.5–3.2 mm long, ellipsoid, of equal width along entire length; corolla barely exceeding the calyx lobe tips before anthesis	***Solanum michaelis* Särkinen & S. Knapp**
12	Anthers 3–3.5 mm long; calyx lobes triangular; leaves narrowly elliptic to lanceolate; stone cells absent	***Solanum profusum* C.V.Morton**
–	Anthers longer than 3.5 mm (occasionally in poorly developed flowers as short as 2.6 mm long), usually 4–5 mm long; calyx lobes narrowly triangular; leaves rhombic to elliptic in outline; stone cells present	**13**
13	Leaf bases truncate, distinctly narrowing to a petiole; anthers ca. 1 mm wide; stone cells 6–8 per berry	***Solanum tweedieanum* Hook.**
–	Leaf bases attenuate onto the petiole and stem, the petiole winged; anthers 1.2–1.5 mm wide; stone cells more than 10 per berry	***Solanum hunzikeri* Chiarini & Cantero**
14	Anthers less than 3 mm long	**15**
–	Anthers more than 3 mm long	**28**
15	Inflorescences forked or several times branched (occasionally with unbranched inflorescences on the same plant, but always some branched)	**16**
–	Inflorescences unbranched	**20**
16	Leaves entire, at most the margins shallowly toothed	**17**
–	Leaves deeply divided or entire and pinnatisect on the same plant	**19**
17	Robust procumbent perennial herbs; berries red; leaves elliptic, the base attenuate; filaments glabrous; anthers ca. 1.7 mm long; currently only known from a local population in Salta	***Solanum tripartitum* Dunal**
–	Variously erect or spreading plants; berries green or purple; leaves ovate to broadly elliptic, with a distinct petiole, the base acute or truncate; filaments with tangled white pubescence adaxially (inside the anther cone); anthers greater than 2 mm long	**18**
18	Stem slightly winged and with spinulose processes; leaf margins toothed, not finely ciliate; pedicels 4–7.5 mm long; style long-exserted from the anther cone, approximately equal to or longer than the anthers; stone cells more than 10 per berry	***Solanum furcatum* Dunal**
–	Stem not winged or with spinulose processes; leaf margins usually entire and finely ciliate, if toothed then still finely ciliate; pedicels more than 8 mm long; style not long exserted from the anther cone, usually shorter than the anthers; stone cells 6–8 per berry	***Solanum zuloagae* Cabrera**
19	Tiny annual herbs; leaves pinnatisect, occasionally with both divided and entire leaves on the same plant, pubescent; corolla pentagonal; calyx accrescent in fruit; mature berry green	***Solanum annuum* C.V.Morton**
–	Robust procumbent perennial herbs; leaves deeply three-parted, glabrous; corolla stellate; calyx not accrescent in fruit; mature berry red	***Solanum tripartitum* Dunal**
20	Tiny annual herbs; corolla pentagonal to rotate; fruiting calyx variously accrescent; seeds tuberculate	**21**
–	Annual or perennial herbs or subshrubs; corolla stellate; fruiting calyx not markedly accrescent; seeds minutely pitted, not tuberculate	**23**
21	Fruiting calyx not enclosing the berry, accrescent but the entire fruit visible; inflorescence with 8–12 flowers; berry with only 2 seeds	***Solanum annuum* C.V.Morton**
–	Fruiting calyx partly to completely enclosing the berry; inflorescence with 2–5 (6) flowers; berry with more than 2 seeds (to 20)	**22**
22	Calyx lobes broadly elliptic to ovate, rounded at the tips, only partially enclosing the berry at maturity; anthers ca. 1 mm long; style only just exceeding the anther cone	***Solanum weddellii* Phil.**
–	Calyx lobes long-triangular, pointed at the tips, inflated and completely enclosing the berry at maturity; anthers usually more than 1 mm long; style exserted from the anther cone	***Solanum gilioides* Rusby**
23	Inflorescences elongate with widely spaced flowers; berries yellow or greenish purple when mature	**24**
–	Inflorescences subumbelliform (with flowers clustered at the tips); berries green or purple when mature	**25**
24	Prostrate herbs with stems often rooting at the nodes; leaves deeply three-parted; corolla rotate; filaments glabrous; berries translucent yellow	***Solanum palitans* C.V.Morton**
–	Erect herbs or subshrubs; leaves entire or shallowly toothed; corolla stellate; filaments pubescent; berries purple or greenish purple when mature	***Solanum furcatum* Dunal**
25	Buds elongate oblong; corolla more than 1 cm in diameter; anthers 2–2.8 mm long, narrowly ellipsoid	**26**
–	Buds ellipsoid to more or less globose; corolla less than 1 cm in diameter; anthers ca. 1 mm long or less, broadly ellipsoid	**27**
26	Mature berry surface matte; stone cells absent; pubescence usually appressed and drying white; peduncle and pedicels strongly deflexed in fruit	***Solanum chenopodioides* Lam.**
–	Mature berry surface shiny; stone cells 2; pubescence spreading; peduncle not deflexed in fruit, the pedicels deflexed and somewhat secund	***Solanum paucidens* Bitter**
27	Stem strongly winged and fleshy; calyx lobes narrowly deltate or triangular; mature berry bright green marbled with white; calyx lobes in fruit spreading, somewhat elongating (to ca. 5 mm long)	***Solanum marmoratum* Barboza & S. Knapp**
–	Stem unwinged, if winged not strongly so, not fleshy; calyx lobes deltate; mature berry black or purplish black; calyx lobes in fruit strongly reflexed, not elongating	***Solanum americanum* Mill.**
28	Leaves deeply divided to pinnatifid, the segments linear or triangular	**29**
–	Leaves entire or shallowly toothed, not deeply divided into distinct lobes	**31**
29	Annual herbs with rooting stems; buds narrowly ellipsoid; anthers ca. 0.5 mm wide, very narrowly ellipsoid	***Solanum triflorum* Nutt.**
–	Perennial plants, the base woody or the stems arising from rhizomes; buds broadly ellipsoid; anthers more than 0.5 mm wide, usually 1 mm wide or wider	**30**
30	Perennial herbs from rhizomes, the base of the plant not markedly woody; leaves completely glabrous; calyx lobes deltate, equal in length to the tube; mature berry pale translucent yellow, with 8 large (more than 1 mm in diameter) stone cells	***Solanum concarense* Hunz.**
–	Subshrubs to shrubs, the base of the plant markedly woody; leaves variously pubescent with appressed simple trichomes; calyx lobes long-triangular to lanceolate, longer than the tube; mature berry green or whitish green, with ca. 10 small (less than 1 mm in diameter) stone cells	***Solanum salicifolium* Phil.**
31	Anthers 3–3.5 mm long, 2–2.5 times longer than wide; buds globose to plump-ellipsoid	**32**
–	Anthers 3.5–6 mm long, 3–6 times longer than wide; buds ellipsoid	**33**
32	Corolla yellow or cream-colored throughout; calyx lobes deltate to broadly triangular; leaf margins not ciliate	***Solanum huayavillense* Del Vitto & Peten.**
–	Corolla white with a green eye; calyx lobes narrowly triangular; leaf margins ciliate	***Solanum zuloagae* Cabrera**
33	Inflorescence branched (forked to many times branched)	**34**
–	Inflorescence unbranched	**38**
34	Fleshy herbs, larger plants sometimes woody at the base; stems decumbent or somewhat erect; leaves glabrous and fleshy; flowers widely spaced on the inflorescence axis; corolla uniformly white; mature berries yellow or pale orange	***Solanum caesium* Griseb.**
–	Shrubs, subshrubs or herbs with woody bases; stems erect; leaves variously pubescent, membranous; flowers closely spaced on inflorescence axis; corolla white or lilac, with a central greenish or yellow-green eye; mature berries green or purple	**35**
35	Pubescence of stems and leaves appressed; stem winged from decurrent leaf bases; fruiting pedicels strongly deflexed	**36**
–	Pubescence of stems and leaves spreading; stem not winged; fruiting pedicels spreading	**37**
36	Pubescence strongly antrorse; inflorescence with 10–20 flowers; calyx lobes 1–2 mm long, deltate with lanceolate tips	***Solanum tiinae* Barboza & S. Knapp**
–	Pubescence appressed but not strongly antrorse; inflorescence with 4–10 flowers; calyx lobes 2.5–3 mm long, long-triangular to lanceolate	***Solanum salicifolium* Phil.**
37	Buds narrowly ellipsoid; corolla deeply stellate, lobed ca. 3/4 of the way to the base; inflorescences generally forked, only rarely more than once branched; berries ca. 0.5 cm in diameter	***Solanum aloysiifolium* Dunal**
–	Buds ellipsoid; corolla stellate, lobed ca. halfway to the base; inflorescences usually many times branched; berries more than 0.5 cm in diameter	***Solanum cochabambense* Bitter**
38	Leaves thick and somewhat fleshy, the margins sharply toothed and often revolute in the sinuses	**39**
–	Leaves thin and membranous, the margins entire or shallowly toothed, never revolute	**42**
39	Buds narrowly ellipsoid; anthers less than 1 mm wide; pubescence of stiff antrorse trichomes; annual herbs	***Solanum triflorum* Nutt.**
–	Buds ellipsoid to broadly ellipsoid; anthers 1 mm wide or wider; pubescence of unicellular papillae or tangled white trichomes, not stiff and antrorse; perennials from a woody base (resprouting from the rhizome every season)	**40**
40	Stems glabrous or with an even covering of minute papillate unicellular trichomes; inflorescence with more than 4 flowers; corolla white or pale violet	***Solanum echegarayi* Hieron.**
–	Stems with pubescence of tangled white multicellular trichomes; inflorescences with fewer than 4 flowers; corolla violet or deep purple	**41**
41	Flowering pedicels 1–2 cm long; calyx lobes acute at the tips; corolla 1–1.2 cm in diameter, deep purple; anthers 4–5.5 mm long; fruiting pedicels 1.5–2 cm long; berry 1–1.5 cm in diameter, bright yellow at maturity	***Solanum sinuatirecurvum* Bitter**
–	Flowering pedicels 0.8–1.1 cm long; calyx lobes rounded at the tips; corolla 1.8–2 cm in diameter, pale lilac or white and lilac; anthers 3.5–4.5 mm long; fruiting pedicels 1.3–1.5 cm long; berry to 1.1 cm in diameter, green or purple	***Solanum riojense* Bitter**
42	Stem with prominent spinulose processes; sympodial units difoliate, the leaves usually geminate; fruiting calyx accrescent and inflated, completely enclosing the berry	***Solanum salamancae* Hunz. & Barboza**
–	Stem terete or angled, without spinulose processes; sympodial units difoliate or plurifoliate, the leaves not geminate; fruiting calyx not accrescent nor completely enclosing the berry	**43**
43	Subshrubs from a markedly woody base; stem angled or very narrowly winged from the decurrent leaf bases; pedicels inserted in enlarged swellings of the inflorescence rhachis, clustered; plants sometimes with entire, toothed and deeply pinnatifid leaves on the same plant	***Solanum salicifolium* Phil.**
–	Herbs, the base of the plant not distinctly woody; stem terete; pedicels not inserted in enlarged swellings from the inflorescence rhachis, spaced or loosely clustered; leaves not markedly variable on the same plant, if variable some leaves with a few basal teeth	**44**
44	Delicate rhizomatous herbs, the stems lax and weak; leaf bases acute to attenuate; leaves elliptic to narrowly elliptic; inflorescence with 2–6 flowers; calyx lobes 1.5–1.8 mm long, narrowly triangular, with acute sinuses; mature berry greyish green	***Solanum pygmaeum* Cav.**
–	Large herbs with sprawling stems, not rhizomatous; leaf bases truncate to somewhat hastate (occasionally slightly cordate); leaves ovate-triangular; inflorescence with 5–15 flowers; calyx lobes 1–1.5 mm long, triangular, with rounded sinuses; mature berry purplish black	***Solanum pilcomayense* Morong**

## Supplementary Material

XML Treatment for Solanum physalidicalyx

XML Treatment for Solanum tweedieanum

XML Treatment for Solanum hunzikeri

XML Treatment for Solanum marmoratum

XML Treatment for Solanum tiinae

## Supplementary materials

Supplementary material 1 SM 1 – all morelloid species Data type occurences Explanation note SM 1 – Specimens of all morelloid species occurring in Argentina. A csv format file of all specimens examined to date with each duplicate as a separate line. Column headings are: Herbarium = herbarium acronym acc. to Index Herbariorum; Category = specimen or photograph of specimen; SpecID = brahms specimen ID; Brahms = Brahms collection event ID; Accession = accession number of specimen; Barcode = barcode of specimen; Collector = principal collector; Prefix = collection number prefix (if applicable); Number = collection number; Suffix = collection number suffix (if applicable); AddColl = additional collectors; Type = type of type; Type of = type of what species; Day -= day of collection; Month = month of collection; Year = year of collection; DateRes = date resolution (if applicable); Family = plant family; Genus = genus name; Species = species epithet; Author = species author name(s); Country = country of collection; Majorarea = first political division; Minorarea = second political division; Gazetteer = nearby town or place; Locnotes = locality; Habitattxt = vegetation characteristics; Lat = latitude; NS = north or south; Longitude = longitude; EW = east or west; LLunit = coordinate units (DD = decimal degrees; DM = decimal minutes; DMS = degrees, minutes, seconds); LLres = coordinate resolution (4 = nearest minute; 7 = area centroid); LLorigin = origin of coordinates; LatLong = coordinates in DMS; LatDec = decimal latitude; LongDec = decimal longitude; Alt = elevation minimum; AltMax = elevation maximum; AltRes = elevation resolution; AltUnit = elevation units (all metres); AltRange = elevational range. https://binary.pensoft.net/file/465509This dataset is made available under the Open Database License (http://opendatacommons.org/licenses/odbl/1.0/). The Open Database License (ODbL) is a license agreement intended to allow users to freely share, modify, and use this Dataset while maintaining this same freedom for others, provided that the original source and author(s) are credited.Sandra Knapp, Franco Chiarini, Juan J. Cantero, Gloria E. Barboza

Supplementary material 2 SM 2– the three new species described here Data type occurences Explanation note SM 2 – Specimens of the new morelloid species described here (*Solanum hunzikeri*, *S. marmoratum* and *S. tiinae*). A csv format file of all specimens examined to date with each duplicate as a separate line. Column headings are as in SM 1. https://binary.pensoft.net/file/465510This dataset is made available under the Open Database License (http://opendatacommons.org/licenses/odbl/1.0/). The Open Database License (ODbL) is a license agreement intended to allow users to freely share, modify, and use this Dataset while maintaining this same freedom for others, provided that the original source and author(s) are credited.Sandra Knapp, Franco Chiarini, Juan J. Cantero, Gloria E. Barboza

## References

[B1] Anton AM Zuloaga FO (2013) Solanaceae. In: Barboza GE (coord.) Flora Argentina (Vol. 13), Solanaceae. IOBDA-IMBIV, CONICET, Buenos Aires & Córdoba, 349 pp.

[B2] Bachman S Moat J Hill AW de la Torre J Scott B (2011) Supporting Red List assessments with GeoCAT: Geospatial conservation assessment tool. ZooKeys 150: 117–126. 10.3897/zookeys.150.2109 PMC3234434 22207809

[B3] Barboza GE Knapp S Särkinen T (2013) Grupo VII. Moreloide. In: Anton AM Zuloaga FO (Eds) Barboza GE (coord.) Flora Argentina (Vol. 13), Solanaceae. IOBDA-IMBIV, CONICET: Buenos Aires & Córdoba, Argentina, 231–264.

[B4] Bitter G (1912) Solana nova vel minus cognita. III. Repertorium Specierum Novarum Regni Vegetabilis 11(9–15): 202–237. 10.1002/fedr.19120110917

[B5] Brown AD (1995) Ecology and conservation of the Argentine montane forest. In: Hamilton LS Juvik JO Scatena FN (Eds) Tropical Montane Cloud Forests.Ecological Studies (Analysis and Synthesis), vol 110. Springer, New York, 107–115. 10.1007/978-1-4612-2500-3_5

[B6] Edmonds JM (1972) A synopsis of the taxonomy of *Solanum* Sect. Solanum (Maurella) in South America. Kew Bulletin 27(1): 95–113. 10.2307/4117874

[B7] Frodin DG (2004) History and concepts of big plant genera. Taxon 53(3): 753–776. 10.2307/4135449

[B8] Hooker WJ (1835) Solanum Tweedianum. Curtis’s Botanical Magazine 62: Tab. 3385.

[B9] IUCN (2019) Guidelines for using the IUCN Red List Categories and Criteria. Version 13. Prepared by the Standards and Petitions Subcommittee. http://www.iucnredlist.org/documents/RedListGuidelines.pdf [accessed: 1 May 2020]

[B10] Knapp S (2013) A revision of the Dulcamaroid clade of *Solanum* L. (Solanaceae). PhytoKeys 22(0): 1–432. 10.3897/phytokeys.22.4041 PMC3689140 23794937

[B11] Knapp S Barboza GE Bohs L Särkinen T (2019) A revision of the Morelloid Clade of *Solanum* L. (Solanaceae) in the Caribbean and North and Central America. PhytoKeys 123: 1–144. 10.3897/phytokeys.123.31738 31198402 PMC6554266

[B12] Morton CV (1976) A Revision of the Argentine Species of *Solanum*. Academia Nacional de Ciencias. Córdoba, 260 pp.

[B13] Moscone EA (1992) Estudios en cromosomas meióticos en Solanaceae de Argentina. Darwiniana 31: 261–297. http://www.jstor.org/stable/23222568

[B14] Philippi RA (1891) Catalogus praevius plantarum in itinere ad Tarapaca a Friderico Philippi lectarum (Catalogus plantarum in itinere Tarapacano lectarum). Anales del Museo Nacional de Chile, Segunda Sección, Botánica 1891 [8]: 1–94. [2 tab.]

[B15] Särkinen T Knapp S (2016) Two new non-spiny *Solanum* (Solanaceae) from the Gran Chaco Americano and a key for the herbaceous glandular-pubescent solanums from the region. PhytoKeys 74: 19–33. 10.3897/phytokeys.74.10159 PMC5234547 28127235

[B16] Särkinen T Olmstead RG Bohs L Knapp S (2013) A phylogenetic framework for evolutionary study of the nightshades (Solanaceae): a dated 1000-tip tree. BMC Evolutionary Biology 13: 214. 10.1186/1471-2148-13-214 PMC3850475 24283922

[B17] Särkinen T Barboza GE Knapp S (2015) True Black nightshades: Phylogeny and delimitation of the Morelloid clade of *Solanum* Taxon 64(5): 945–958. 10.12705/645.5

[B18] Särkinen T Poczai P Barboza GE van der Weerden GM Baden M Knapp S (2018) A revision of the Old World black nightshades (Morelloid clade of *Solanum* L., Solanaceae). PhytoKeys 106: 1–223. 10.3897/phytokeys.106.21991 PMC6070582 30072843

[B19] Spooner DM Jansky S Rodríguez F Simon R Ames M Fajardo D Castillo RO (2019) Taxonomy of wild potatoes in northern South America (Solanum section Petota). Systematic Botany Monographs 108: 1–305.

[B20] Turland NJ Wiersema JH Barrie FR Greuter W Hawksworth DL Herendeen PS Knapp S Kusber W-H Li D-Z Marhold K May TW McNeill J Monro AM Prado J Price MJ Smith GF (2018) International Code of Nomenclature for algae, fungi, and plants (Shenzhen Code) adopted by the Nineteenth International Botanical Congress, Shenzhen, China, July 2017. Regnum Vegetabile 159. Koelz Botanical Books, Gläshutten. 10.12705/Code.2018

